# Mechanistic Insights of a p53-Targeting Small Molecule

**DOI:** 10.1021/acsptsci.5c00110

**Published:** 2025-05-07

**Authors:** Ricardo J. F. Ferreira, Valentina Barcherini, Catarina Roma-Rodrigues, Miriama Sikorová, Lucília Saraiva, Ana P. Leandro, Pedro V. Baptista, Alexandra R. Fernandes, Alexandra M. M. Antunes, Maria M. M. Santos

**Affiliations:** ! Research Institute for Medicines (iMed.ULisboa), Faculty of Pharmacy, 451167 Universidade de Lisboa, Av. Prof. Gama Pinto, Lisboa 1649-003, Portugal; @ Associate Laboratory i4HB—Institute for Health and Bioeconomy, NOVA School of Science and Technology, 70887 NOVA University Lisbon, Caparica 2819-516, Portugal; # UCIBIO—Applied Molecular Biosciences Unit, Department of Life Sciences, NOVA School of Science and Technology, NOVA University Lisbon, Caparica 2819-516, Portugal; $ BIORG—Bioengineering and Sustainability Research Group, Faculdade de Engenharia, Universidade Lusófona, Lisboa 1749-024, Portugal; % LAQV/REQUIMTE, Laboratório de Microbiologia, Departamento de Ciências Biológicas, Faculdade de Farmácia, Universidade do Porto, Rua de Jorge Viterbo Ferreira 228, Porto, 4050-313, Portugal; & Centro de Química Estrutural (CQE), Institute of Molecular Sciences, Departamento de Engenharia Química, Instituto Superior Técnico (IST), Universidade de Lisboa, Lisboa 1049-001, Portugal

**Keywords:** p53, protein modification, cancer, isoindolinones, cysteine

## Abstract

Restoring the p53 pathway, particularly by reactivating wild-type (wt) or mutant (mut) p53, is considered a promising approach for cancer treatment. Previously, we identified the tryptophanol-derived oxazoloisoindolinone family as a new scaffold for obtaining wt and mut p53 reactivators. Herein, we report a detailed study on the pharmacokinetic profile and the mechanism of action of **RVJB59**, an (*R*)-tryptophanol-derived oxazoloisoindolinone that exhibits six times higher activity and increased selectivity for HCT116 cells with p53 compared to our initial hit, **SLMP53–1**. *In vitro* metabolic degradation studies in human liver microsomes and rat liver S9 fractions, assessed by LC/HRMS/MS, showed that **RVJB59** is a low-clearance compound. The three main metabolites identified were synthesized, and their antiproliferative activity was evaluated against HCT116 colon cancer cells with and without p53, showing a loss of activity when compared to **RVJB59**. DSF studies showed that **RVJB59** enhances the thermostability of the wt and R273H p53 DNA-binding domain, with this mutant showing melting curves with two melting transitions, distinct from those obtained for the wild-type. The ability of **RVJB59** to undergo covalent binding via nucleophilic aromatic substitution was assessed by HRMS/MS, using glutathione and wt p53 as case studies. These assays showed low reactivity toward glutathione and remarkable selectivity toward Cys141 of wt p53. The effect of **RVJB59** was also evaluated in 3D spheroids of HCT116 cells and *in vivo* using chicken embryos, with **RVJB59** reducing 3D tumor spheroid growth and exhibiting antiangiogenic potential. This study provides additional evidence of the potential of **RVJB59** in activating p53.

## Graphical abstract

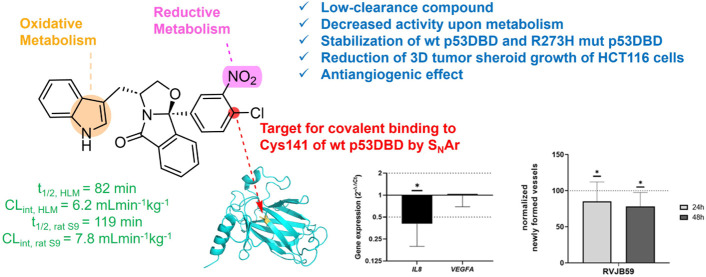

## 1 Introduction

The tumor suppressor protein p53 is a DNA-binding transcription factor with a key role in cell fate. In response to different stress signals, p53 acts as a tumor suppressor protein, acting as a major barrier against cancer. By activating or repressing the expression of target genes, p53 can lead to cell cycle arrest, apoptosis, DNA repair, senescence, or autophagy processes.^1^ The p53 protein is composed of 393 amino acids and comprises multiple structural and functional domains.^2^ The p53 DNA-binding domain (p53DBD) is responsible for p53’s transcriptional activity and the binding of the protein to specific DNA sequences. Previous studies showed that isolated p53DBD, without its oligomerization domain, exists as a monomer in solution and is active for DNA binding *in vitro,* even in the absence of the *C*-terminal domain.^3,4^

In human cancers, p53 is commonly found inactivated either by mutation of the *TP53* gene or by upregulation of its negative regulators. Moreover, the *TP53* gene is the most frequently mutated gene in human cancer, occurring in approximately 50% of all human tumors. These mutations are often associated with resistance to anticancer drugs and poor overall survival. So, the development of small molecules capable of reactivating the p53 tumor suppressor function in this type of cancer is highly needed.

The most common strategies to achieve this goal are the inhibition of the main p53 negative regulators, MDM2 or MDM4, and reactivating p53 wild-type (wt) functions in cancers with mutated p53.^5,6^ Currently, several MDM2 inhibitors are in clinical trials, but none has yet reached the clinic (Figure 1). However, there are still very few small molecules able to reactivate the wild-type functions of the mutant (mut) p53 protein that have entered clinical trials. These include (Figure 1), the cysteine-alkylating pro-drug **Eprenetapopt** (also known as **APR-246**), **arsenic trioxide**, and **Rezatapopt** (also known as **PC14586**). **APR-246** restores the wild-type p53 conformation and function, which subsequently binds to DNA, inducing apoptosis in human cancer cells. *In vivo*, **APR-246** is converted into the Michael acceptor methylene quinuclidinone (**MQ**), which binds to the most exposed cysteine residues present in the DBD of wild-type and mutated p53. **APR-246** has shown preclinical antitumor activity in a wide variety of solid and hematological cancers, as well as strong synergy with anticancer drugs.^7^ **Arsenic trioxide** is capable of rescuing transactional activity in several p53 mutants.^8,9^ **PC14586** binds to the p53 Y220C mutant, restoring the p53 wild-type conformation and transcriptional activity, leading to potent preclinical antitumor activity.^8,10^

**Figure 1:**
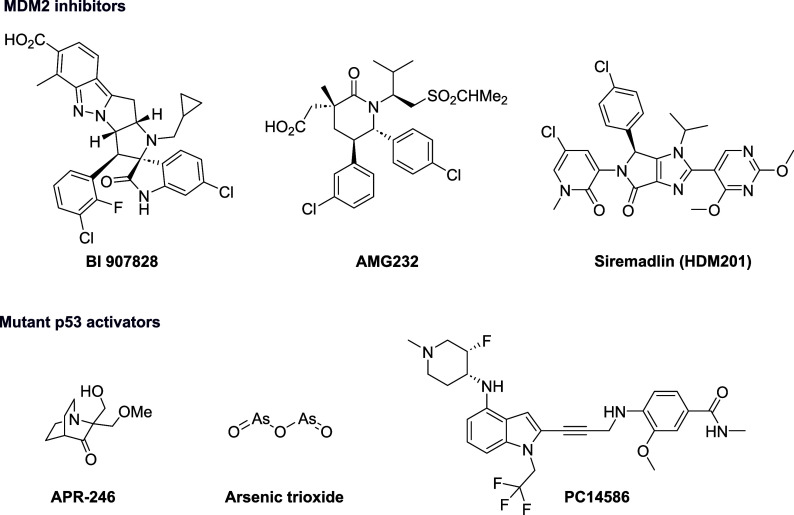
Selected examples of p53 activators that have entered clinical trials.

In previous work, the enantiopure tryptophanol-derived oxazoloisoindolinone **SLMP53–1** was identified as a direct reactivator of wt p53 and the DNA-contact mutant R280 K p53 with *in vitro* and *in vivo* p53-dependent antitumor activity.^11,12^ A structure–activity relationship (SAR) study was conducted, leading to the identification of **SLMP53–2**, a reactivator of the structural mutant Y220C p53,^13^ and **RVJB59**, a small molecule 6-fold and 3.2-fold more active than the hits **SLMP53–1** and **SLMP53–2**, respectively, and with increased preference for HCT116 p53^+/+^ cells over HCT116 p53^–/–^ cells (Figure 2).^14^

**Figure 2:**
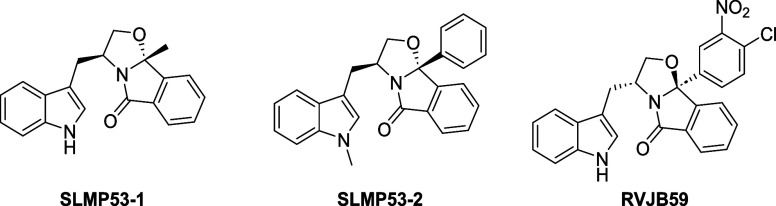
Chemical structures of tryptophanol-derived oxazoloisoindolinones **SLMP53–1**, **SLMP53–2**, and **RVJB59**.

To extend our knowledge about the pharmacokinetic (PK) profile and the mechanism of action of compound **RVJB59**, we have identified the compound’s metabolites and evaluated the possible interaction of this small molecule with wt p53DBD through an *in vitro* differential scanning fluorimetry (DSF) assay. By high-resolution mass spectrometry (HRMS), we identified the selective covalent binding of **RVJB59** to Cys 141 of wt p53DBD.

## 2 Results and Discussion

### 2.1 Metabolic Stability of Compound **RVJB59**

In our previous PK studies, we observed that the tryptophanol-derived oxazoloisoindolinones **SLMP53–1** and **SLMP53–2** are low-clearance compounds, with half-lives of 138 and 147 min, respectively. The indole ring was identified as the primary target for metabolic transformations of these derivatives, yielding less active indole ring-opening metabolites via oxidative metabolic pathways.^15^

Due to the potential soft metabolic nature of nitro substituents, the metabolic effects of this substituent in **RVJB59** were investigated *in vitro*. Despite a slight decrease in metabolic stability compared to **SLMP53–1** and **SLMP53–2**, **RVJB59** remains a low-clearance compound,^16^ exhibiting a half-life of 82 min (CL_int_ 6.2 mLmin^–1^ kg^–1^) in human liver microsomes (HLM) (Figure S1). Similar results were obtained in rat S9 liver fraction incubations (half-life of 119 min, CL_int_ = 7.8 mLmin^–1^ kg^–1^) (Figure S2). These results suggest that cytosolic enzymes do not have a major contribution to the metabolic degradation of **RVJB59**. In addition, it was observed that the potential toxic nitroreduction pathway^17^ is not a primary metabolic route for **RVJB59**, as the highest nitroaromatic reduction activities were reported to be observed in rat cytosolic fractions.^18^

Very similar metabolite profiles were observed in both HLM and rat liver S9 fractions, with the indole ring consistently being the primary target for the metabolic degradation of **RVJB59** (Scheme 1 and Figure S3). The primary metabolic transformation involved oxidation at the indole C-2 position, resulting in the formation of the two oxindole diastereomers, **M1** (Figure S4). Minor metabolic products included the formamide metabolite **M2** (Figure S5), formed through multiple oxidative steps with the concomitant ring opening of the indole pyrrole, and **M3** (Figure S6), stemming from the reduction of the nitro group (Scheme 1). Notably, although the reductive metabolism of nitroaromatic moieties often leads to potentially toxic nitrenium intermediates, the bioreductive metabolism of compound **RVJB59** constitutes only a minor pathway.

**Scheme 1:**
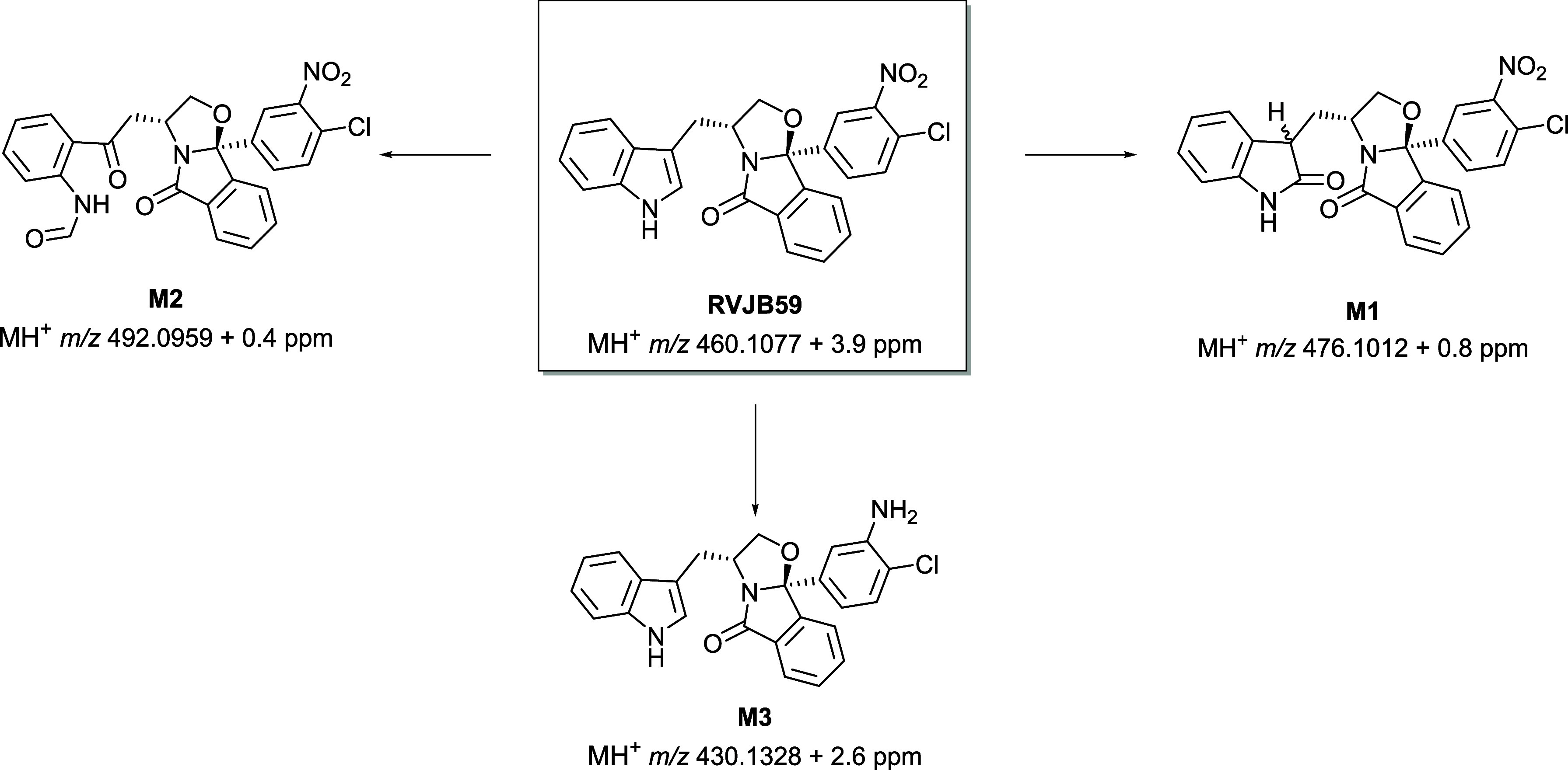
Metabolite Profile of **RVJB59** in HLM and Rat Liver S9 Fraction Incubations, Obtained by LC-HRMS/MS Analysis upon Comparison with Synthetic Standards

Liquid chromatography tandem high-resolution mass spectrometry (LC-HRMS/MS), upon comparison with fully characterized synthetic standards, was used for unequivocal metabolite identification. **M1** and **M2** standards were obtained by biomimetic catalysis, by a methodology similar to the one previously used for the metabolite synthesis of other tryptophanol-based derivatives (Scheme 2).^11^

**Scheme 2:**
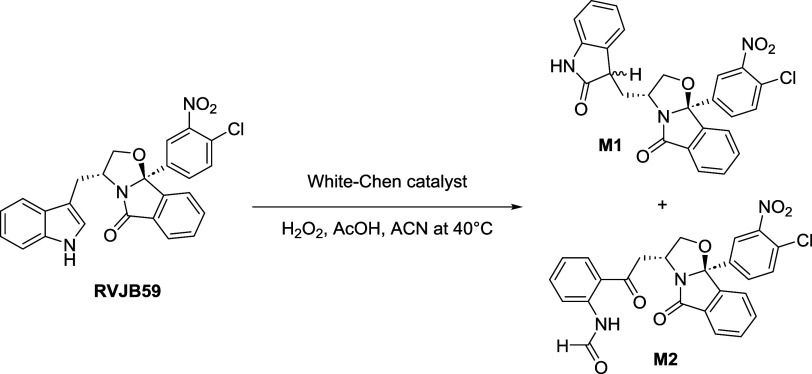
Biomimetic Oxidation of **RVJB59** for the Preparation of **M1** and **M2** Standards

Metabolite **M3** was prepared in a 76% yield by a cyclocondensation reaction of (*R*)-tryptophanol with 2-(4-chloro-3-aminophenyl)­benzoic acid (Scheme 3).

**Scheme 3:**
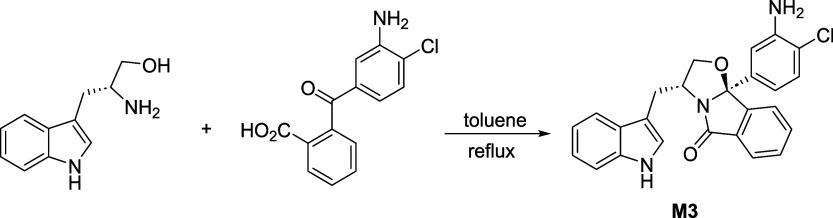
Synthesis of Tryptophanol-Derived Oxazoloisoindolinone **M3**

### 2.2 Antiproliferative Activity of **RVJB59** and Its Major Phase I Metabolites

The antiproliferative activity of **M1**, **M2**, and **M3** metabolites was evaluated in the isogenic pair of HCT116 cancer cells with and without p53 (Table 1). The major metabolite **M1** was much less active than **RVJB59** in both cell lines, while the indole ring-opening **M2** and the amino-derived **M3** metabolites were only 2.3- and 2.0-fold less active than the parent compound **RVJB59** in HCT116 p53^+/+^ cells, respectively. The obtained results suggest that, although secondary, the oxidative and bioreductive metabolism of compound **RVJB59** may have a pharmacological impact toward the tumor suppressor p53 pathway. In addition, these results show that the activity observed for **RVJB59** is not due to its metabolites, indicating that **RVJB59** does not function as a pro-drug.

**Table 1:** Antiproliferative Activities of **RVJB59** and Its Metabolites^a^

Entry	Compounds	HCT116 p53^+/+^ GI_50_ (μM)^b^	HCT116 p53^–/–^ GI_50_ (μM)^b^	SI
1	**M1**	44.35 ± 4.65	>50	ND
2	**M2**	8.85 ± 0.05	18.6 ± 3.0	2.1
3	**M3**	7.55 ± 0.35	11.5 ± 0.5	1.5
4	**RVJB59**	3.85 ± 0.15	9.95 ± 0.05	2.6

a ND – not determined.

b Results are reported as growth inhibition values (GI_50_) determined by SRB assay. Results are reported as mean ± SEM and correspond to 5 independent experiments.

The antiproliferative activity of **RVJB59** was then assessed in HT29 cancer cells expressing the R273H mutant p53. The GI_50_ of 15 μM in HT29 cells (Table 2) suggests that **RVJB59** may restore the p53 functions of the R273H mutant p53. Finally, **RVJB59** was also tested in normal human fibroblasts to evaluate the selectivity of the molecule for cancer cells (Table 2). For the tested concentrations, **RVJB59** was not active against human fibroblasts, indicating that it is not toxic in normal cells at the GI_50_ concentration in HCT116 p53^+/+^ and in HT29 R273H mutant p53 cells.

**Table 2:** Antiproliferative Activity of **RVJB59** in HT29 Cells and Fibroblasts

Compound	HT29 GI_50_ (μM)^a^	Fibroblasts GI_50_ (μM)^a^
RVJB59	15.2± 1.1	>50

a Results are reported as growth inhibition values (GI_50_) determined by the MTS assay.

### 2.3 **RVJB59** Enhances the Thermostability of the Wild-Type p53 DNA-Binding Domain

To further evaluate the mechanism of action of compound **RVJB59**, the compound was tested against wt p53DBD using differential scanning fluorimetry (DSF). In this assay, an increase in the p53DBD melting temperature (*T*_m_) indicates stabilization of the protein upon binding of the ligand (Figure 3).^19^ The active form of **APR-246**, methylene quinuclidinone (**MQ**), was used as a positive control.^20–22^ When incubated with **MQ** (2 mM), the wt p53DBD presented a *T*_m_ of 42.45 ± 0.23 °C, corresponding to an increase of 2 °C (Figure S7), while when incubated with **RVJB59** (2.5 mM), the calculated *T*_m_ was 41.89 ± 0.06 °C, corresponding to an increase of 1.43 °C. These results suggest that **RVJB59** could interact directly with wt p53DBD, enhancing its thermostability.

**Figure 3:**
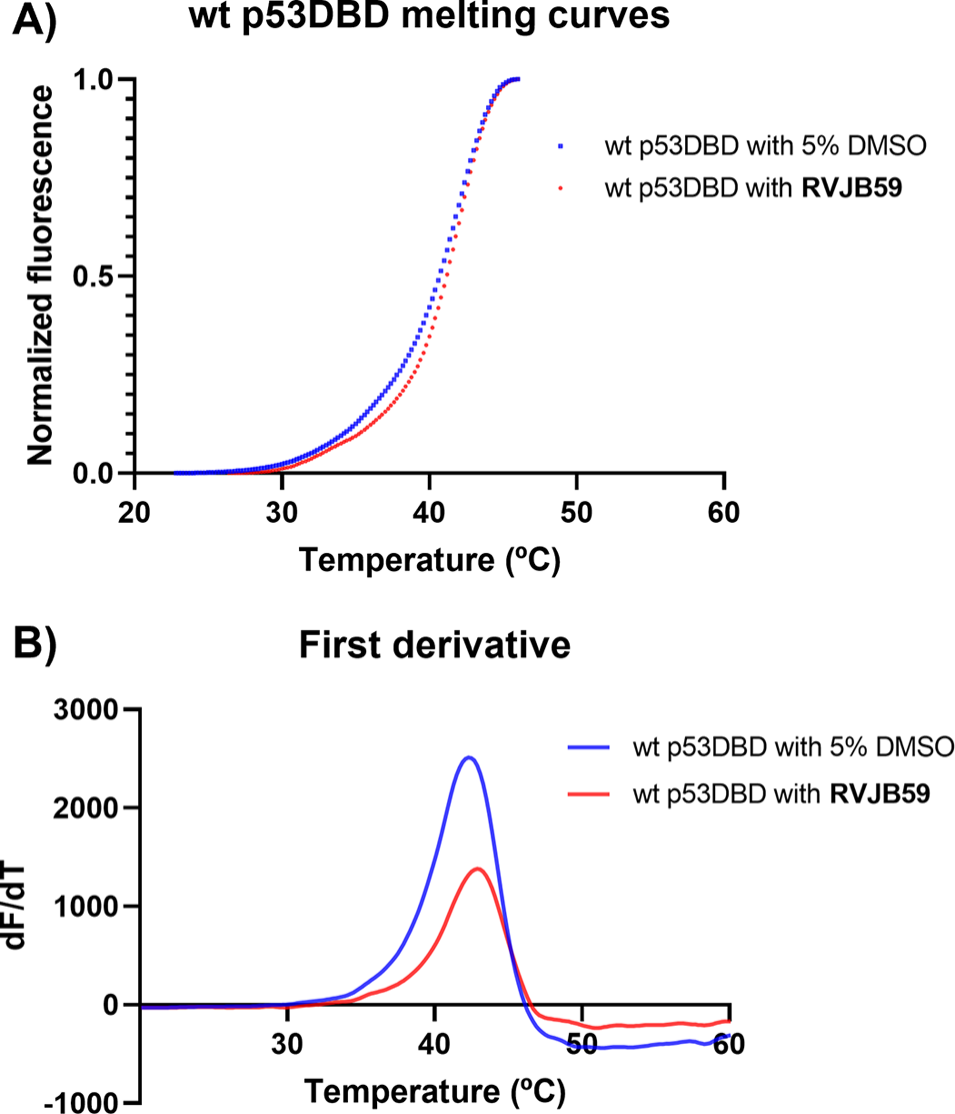
(A) Thermal denaturation curves were obtained by DSF assay of the wt p53DBD in the absence and presence of **RVJB59** (2.5 mM). (B) First order derivative (d*F*/d*T*) of melting curves of the wt p53DBD in the absence and presence of **RVJB59** (2.5 mM). The DSF assays were performed with the fluorophore SYPRO Orange.

The antiproliferative assays with the HT29 cell line previously suggested the possible restoration of mutant p53 functions by **RVJB59**. To evaluate this hypothesis, the R273H mut p53DBD was produced, and its stabilization by **RVJB59** was also evaluated using a DSF assay. In the absence of the compound, a monophasic melting curve was obtained, for which a *T*_m_ of 36.10 ± 0.17 °C was determined (Figure 4), indicating a protein with lower stability compared to the wt. However, in the presence of **RVJB59** (2.5 mM), the melting curve showed a denaturation profile different from that obtained for wt p53DBD in the presence of **RVJB59**, with two denaturation transitions (Figure 4) corresponding to the *T*_ms_ of 34.91 ± 0.06 °C and 63.55 ± 0.50 °C. More detailed studies of the interaction between **RVJB59** and R273H mut p53 need to be carried out in the future to understand this behavior.

**Figure 4:**
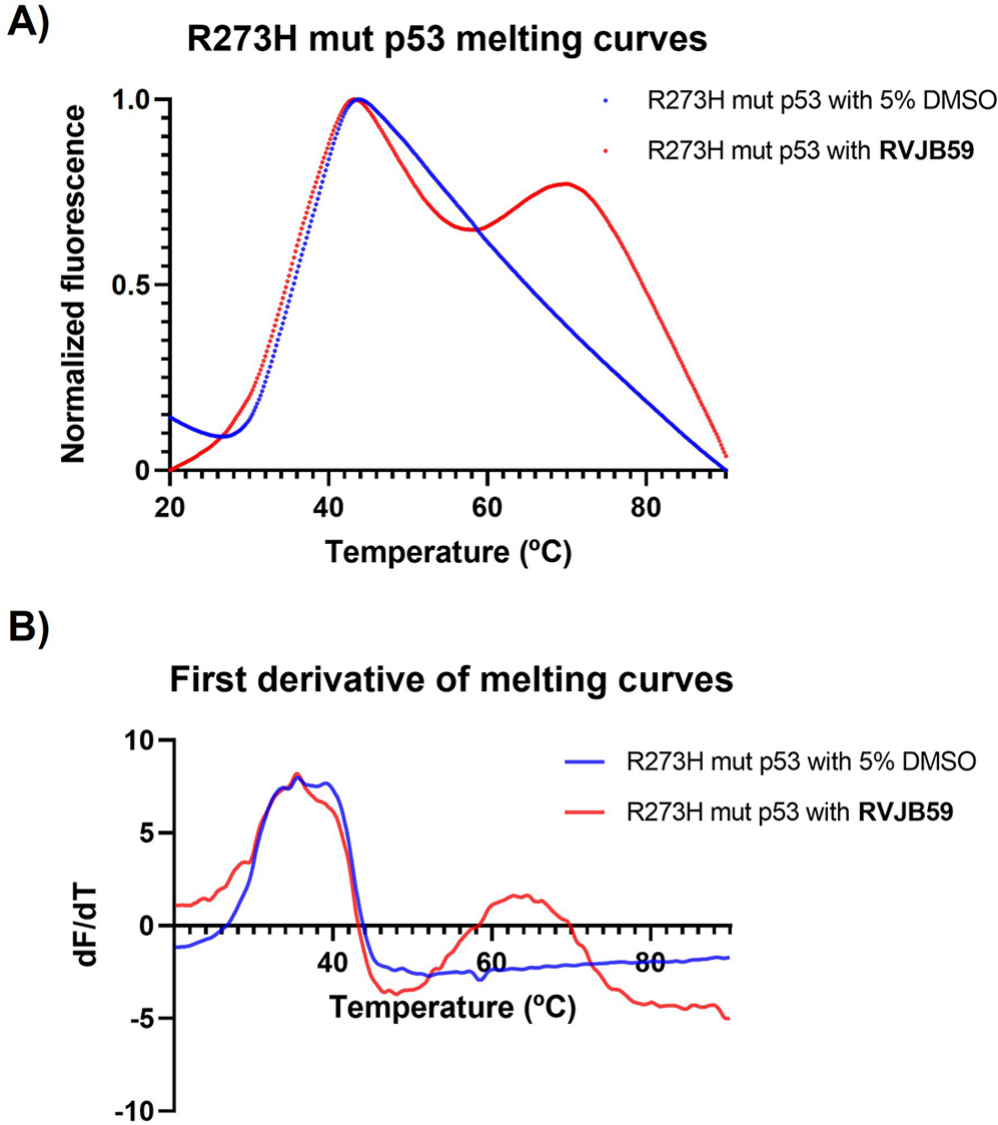
(A) Thermal denaturation curves obtained by DSF assay of the R273H mut p53DBD in the absence and presence of **RVJB59** (2.5 mM). (B) First order derivative (d*F*/d*T*) of melting curves of the R273H mut p53DBD in the absence and presence of **RVJB59** (2.5 mM). The DSF assays were performed with fluorophore SYPRO Orange.

### 2.4 Covalent Modification of p53 by **RVJB59**

In the past decade, we have witnessed a resurgence in the development of covalent drug candidates, owing to their pharmacological advantages.^23–25^ One illustrative example is the wt-p53 activator **APR-246**, which is bioactivated to the Michael acceptor methylene quinuclidinone (**MQ**), which reacts with cysteines in the p53 core domain. Due to the presence of a nitro group *ortho* to a chlorine atom in the phenyl ring substituent in **RVJB59**, we evaluated whether **RVJB59** could be a target for nucleophilic aromatic substitution by bionucleophiles.^26,27^ This hypothesis was first confirmed through *in vitro* incubation of **RVJB59** with glutathione (GSH), which yielded an HRMS signal consistent with the substitution of chlorine by GSH, as indicated by the loss of the Cl isotopic pattern. The identification of the corresponding fragment ions in the MS/MS spectrum (Figures 5 and S8) further supported this observation. These results suggest that **RVJB59** could covalently modify cysteine residues in wild-type p53 without requiring prior bioactivation. Additionally, the kinetics of the reaction with GSH were evaluated using **MQ** and **acrylamide** as controls. Interestingly, while **MQ** and **acrylamide** glutathione adducts were observed in the initial time points of the reaction, with 50% conversion observed after 150 min, only residual formation of **RVJB59**-glutathione was observed at this time point. These observations suggest higher bioavailability and lower off-target reactions for **RVJB59** when compared with the model compounds **MQ** and **acrylamide**.^28^

**Figure 5:**
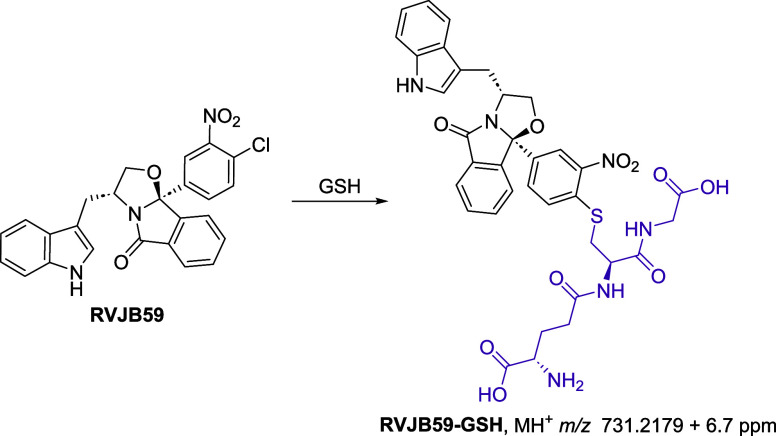
Formation of **RVJB59**-glutathione adduct via nucleophilic aromatic substitution, which was characterized by HRMS/MS.

Considering the evidence of **RVJB59**’s ability to covalently modify glutathione, we decided to investigate whether it could also modify proteins and used wt p53DBD as a case study. For that, **RVJB59** was incubated with wt p53DBD in 1:1 and 1:4 molar ratios (p53 protein:**RVJB59**), followed by digestion with trypsin. **MQ** was used as a positive control (Figures S11 and S12). The subsequent analysis by LC-HRMS/MS allowed the identification of Cys141 bearing a mass increment compatible with the incorporation of **RVJB59**, with the concomitant loss of the chlorine substituent (HRMS/MS spectra of tri- and dicharged ions are displayed in Figures S13 and S14, respectively). This mass increment was exclusively detected in Cys141, highlighting a remarkable selectivity toward this residue. Cys141 has previously been identified as a primary p53 site of modification for other covalent modifiers^29^ and is associated with conformational instability/plasticity of the human p53 DBD.^30^ However, with other covalent modifiers, it was suggested that Cys141 is only made accessible upon structural alteration caused by initial covalent modification of the more accessible Cys124 residue.^29^ This is also consistent with our observation of two MQ modifications at Cys124 and Cys141 under the same incubation conditions used for **RVJB59** modification (Figures S11 and S12). Therefore, a plausible explanation for **RVJB59** selectivity could be its initial noncovalent interaction, which might facilitate the conformational change necessary to expose Cys141 for covalent modifications. While the chemical space of covalent modifiers has expanded significantly in recent years, Michael acceptors remain the most employed in covalent drug strategies, with very few examples of nucleophilic aromatic substitution as a covalent modification strategy in drug design.^24,26,27,29,31^ Moreover, we would like to highlight not only the remarkable selectivity of **RVJB59** toward p53 Cys141 but also its very slow reaction kinetics with glutathione, which consistently suggest its minimized off-target reactivity.

### 2.5 3D Spheroid Growth and Invasion Assays

The activity of **RVJB59** in 3D spheroids of colorectal cancer cells was also evaluated. Spheroids of HCT116 cells were prepared in ultralow-attachment U-bottom 96-well plates and allowed to grow until the fourth day. On this day, 50 μL of medium was replaced by 50 μL of high-concentration Matrigel basement membrane (Corning, Merck) mixed 1:1 with 21.1 μM of **RVJB59**, 20 ng/mL endothelial growth factor (EGF, positive control), or 0.4% (v/v) DMSO (negative control), according to procedures previously described.^32,33^ This concentration of **RVJB59** was selected to ensure good drug diffusion within the 3D tumor spheroid. Images were acquired on the 11th day (7 days after Matrigel supplementation), and the average diameter was measured. Interestingly, spheroids treated with **RVJB59** attained a smaller size (a reduction of 13% in size) compared to DMSO-treated spheroids (Figure 6). Contrary to **RVJB59** and as expected, the epithelial growth factor was able to stimulate spheroid growth (an increase of 14.9% in size).

**Figure 6:**
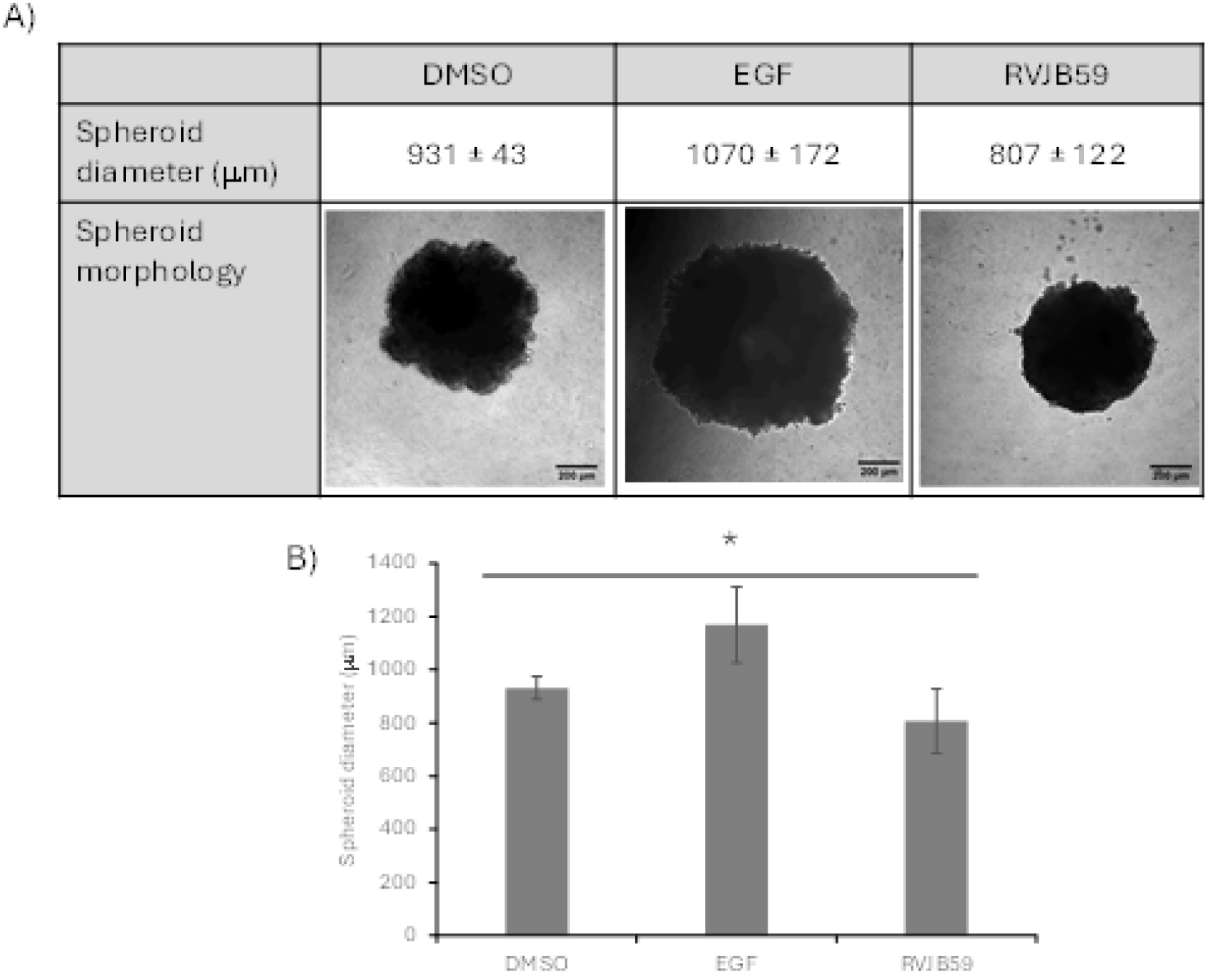
Spheroids growth in the presence of Matrigel supplemented with compound **RVJB59**. HCT116 spheroids cultured for 4 days, were embedded to Matrigel in medium supplemented with 21.1 μM **RVJB59**, 20 ng/mL endothelial growth factor (EGF, positive control), or 0.1% (v/v) DMSO (vector control). Scale bars represent 200 mm. (A) Representative images of the spheroids, acquired after 11 days old (7 days after Matrigel supplementation), were obtained, and the diameter of the spheroid was measured. (B) Spheroid size difference between **RVJB59-**, EGF-, and DMSO-treated HCT116 spheroids. Bars represent the average and standard deviation of at least 3 independent experiments. *p-value <0.05.

The following step was to understand whether the compound influenced the growth of multicellular spheroids composed of colorectal cancer HCT116 cells and fibroblasts, which are important players in the tumor microenvironment. In fact, heterotypic spheroids composed of HCT116 cancer cells and fibroblasts are a better model for studying TME than homotypic spheroids, highlighting the relevance of fibroblasts in the modulation of spheroid growth, viability, hypoxia, and inflammation. Hence, HCT116 and HCT116-fibroblast spheroids were prepared according to procedures previously described^34^ and incubated with 21.1 μM of **RVJB59**, EGF, or 0.1% DMSO (control) after 4 days of growth. Images were acquired every 24 h, and the average diameter of each spheroid and the growth rate of the spheroids during the subsequent 4 days were calculated. Results showed that HCT116-fibroblast spheroids grow slower than homotypic spheroids. Additionally, exposure of spheroids to **RVJB59** led to a reduction in their growth compared to spheroids exposed to the DMSO control, while spheroids exposed to EGF induced faster growth. This is particularly relevant for heterotypic spheroids, where **RVJB59** reduced the growth rate from 6.8 ± 0.8 μm/day in the DMSO control to 4.8 ± 1.5 μm/day, whereas EGF increased the growth rate to 12.7 ± 1.0 μm/day (Figure 7). The reduction of the spheroid growth rate induced by **RVJB59** agrees with the results presented in Figure 6 and highlights the relevance of this small molecule in avoiding the growth of both homotypic and heterotypic tumor spheroids.

**Figure 7:**
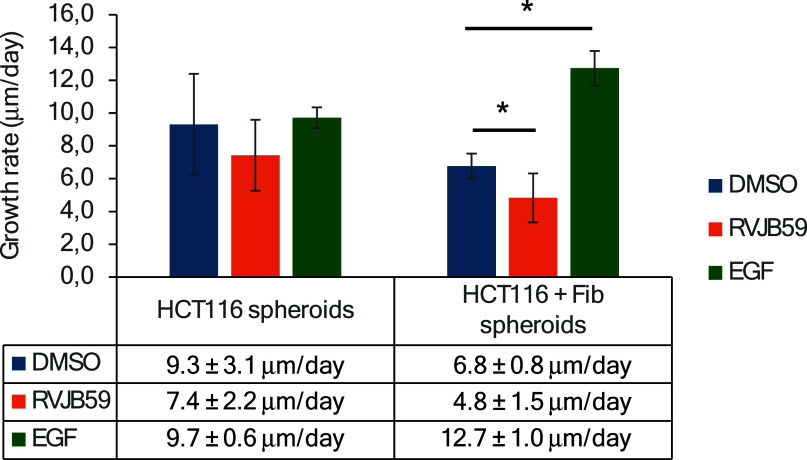
Spheroids growth rate in the presence of **RVJB59**, EGF, or DMSO. HCT116 spheroids and HCT116+ fibroblast spheroids were grown for 4 days and after incubated with medium supplemented with 21.1 μM **RVJB59**, 20 ng/mL of endothelial growth factor (EGF, positive control), or 0.1% (v/v) DMSO (vector control). Images were acquired every 24 h for 4 additional days and both the spheroid diameter and the growth rate of the spheroid were calculated. *p-value <0.05.

Another important process in tumorigenesis is the ability of cells to migrate, leading to cancer metastasis.^35^ In this context, the migration of tumor cells toward capillary veins and the migration of endothelial cells to form new vessels are relevant in this process.^35^ Hence, the ability of HCT116 cancer cell lines and the endothelial cell line human umbilical vein endothelial cells (HUVEC) to migrate in the presence of the compound **RVJB59** was analyzed. A scratch was performed on HCT116 and HUVEC 2D monolayers, which were then incubated for 24 h with 21.1 μM of **RVJB59** or 0.1% (v/v) DMSO (control). The average size of the scratch was measured at 0 and 24 h, and the percentage of wound remission was calculated. Results showed that, despite a slight reduction in the percentage of remission observed in HCT116 cells, no statistically significant difference was observed, suggesting that the compound does not provide an inhibitory effect on the migration ability of cancer cells (Figures 7A and 8C). Nevertheless, a decrease in the percentage of remission was observed when HUVEC cells were exposed to **RVJB59** compared to the control (Figures 7B and 8C).

**Figure 8:**
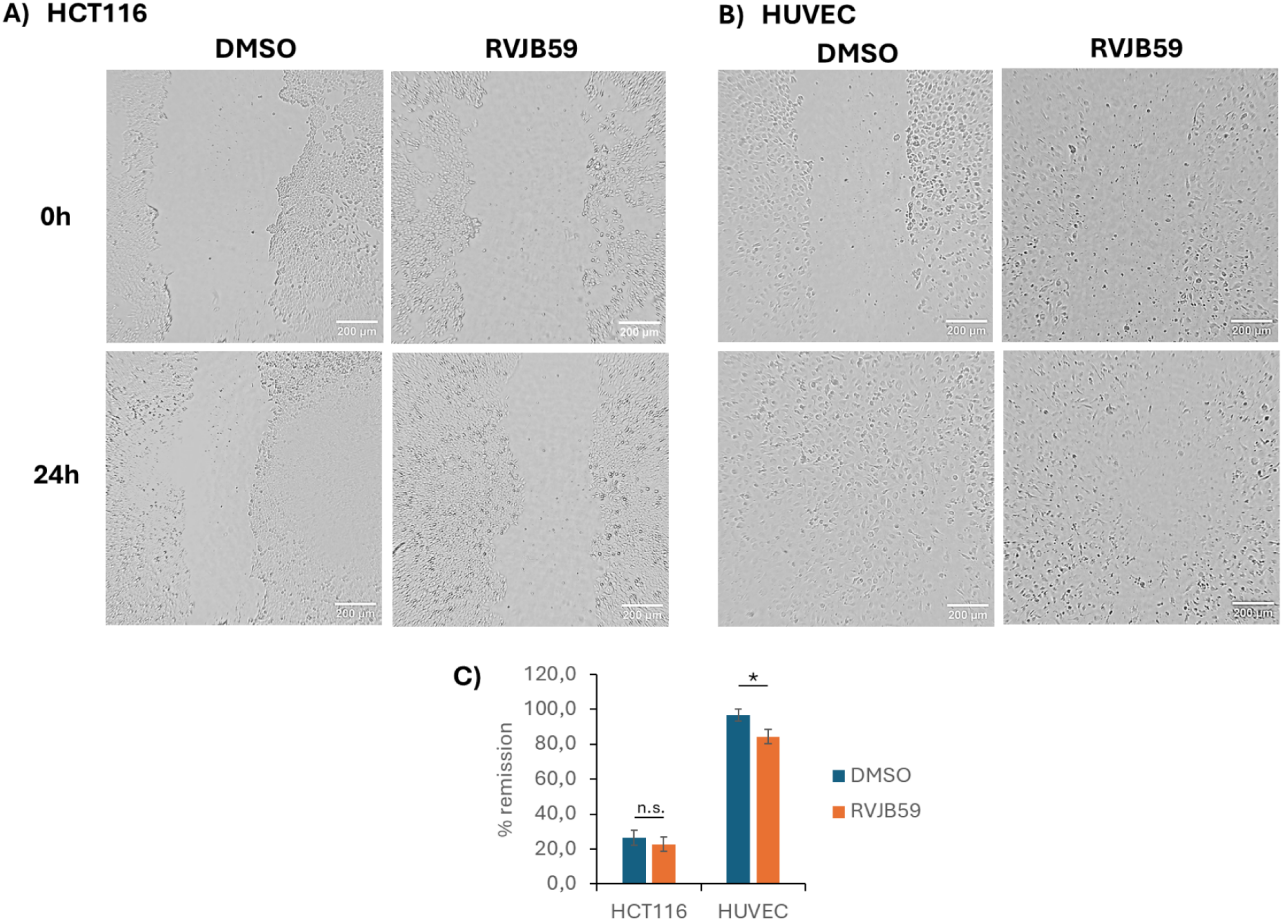
Wound healing assay. Representative images of the wound healing assay at 0 h and 24 h after incubation with 0.1% (v/v) DMSO (control) or 21.1 μM of **RVJB59** in (A) HCT116 and (B) HUVEC. Scale bars correspond to 200 μm. (C) Percentage of wound scratch closure (% remission) after 24 h of incubation with 0.1% (v/v) DMSO (control) or 21.1 μM of **RVJB59**. Bars represent the mean ± SEM of at least two independent experiments. n.s. – nonstatistically significant.

To determine whether the effect of **RVJB59** in HUVEC extends to the angiogenic process, their ability to form capillaries was analyzed according to procedures previously described.^36^ For this purpose, HUVEC were mixed with 21.1 μM of **RVJB59** or 0.1% (v/v) DMSO, placed on top of Matrigel, and the development of capillary-like structures was monitored every 15 min throughout the course of 8 h using a LUX2 microscope (Axion Biosystems). Results showed that capillary-like tubes began to form after 1h and 30 min in DMSO-treated samples but only after 2 h 30 min in **RVJB59**-treated samples, corresponding to a delay of 1 h in samples exposed to the compound compared to the control (Figure 8). However, tube degradation was observed after 5 h in both samples (Figure 9).

**Figure 9:**
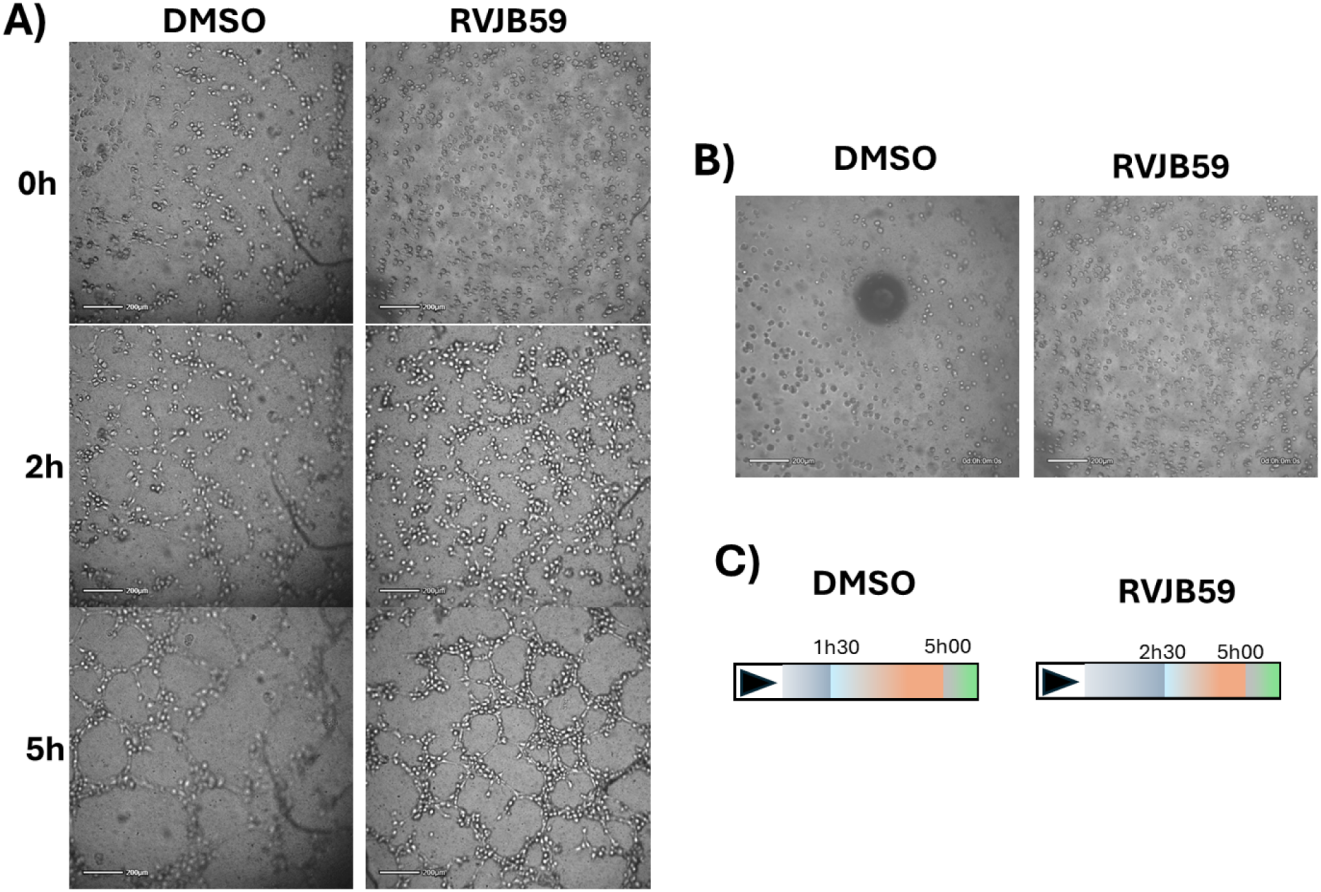
Capillary-like tube formation by a HUVEC. (A) Representative images of cells after 0, 2, and 5 h of cells seeding on top of Matrigel in F12–K medium supplemented with 0.1% (v/v) DMSO (vector control) or 21.1 μM **RVJB59**. Scale bar corresponds to 200 μm. (B) Development of tube formation throughout time. (C) Schematics of tube development throughout time. The end of the blue bar represents the time point where the tube formation starts, and the end of the orange bar represents the maximum tube formation, where the tube degeneration starts (gray bar).

Additionally, considering all these previous results, the antiangiogenic capacity of compound **RVJB59** was also assessed based on an *in vivo* assay with chicken embryos, as previously described.^37^ Fertilized eggs were incubated for 72 h at 38 °C and 90% (v/v) humidity, then opened, and stabilized for an additional 6 h. Then, black silicone O-rings were distributed in the blood vessels of the yolk sack in an equitable distance (Figure 10A), and 21.1 μM of **RVJB59**, 0.1% DMSO (vehicle control), or PBS were added to each O-ring. The internal area of each O-ring was imaged at 0 h, and again after 24 h and 48 h (Figure 10B), and analyzed^37^ to calculate the percentage of newly formed vessels (Figure 10C). Results showed that compound **RVJB59** treatment resulted in a decrease of newly formed vessels, observed after 24 h and maintained after 48h (Figure 10C), supporting the previous results (Figures 7 and 8) that suggested the antiangiogenic potential of the compound. To better understand the pathways involved in the inhibition of new capillary formation, the expression of genes involved in the angiogenic process was evaluated by extracting RNA from the chicken embryos after 12 h of incubation with 21.1 μM of **RVJB59** or 0.1% (v/v) DMSO and evaluate the relative expression of *IL8* and *VEGFA* according to procedures already described.^38^ Interestingly, while the expression of *VEGFA* was similar in both control and **RVJB59**-treated embryos (Figure 10D), a decrease in the expression of *IL8*, an inflammatory cytokine, was observed in **RVJB59**-treated embryos. Interestingly, a correlation between aberrant p53 and increased *IL8* expression was previously described in nonsmall-cell lung cancer patients.^39,40^ Our results suggest that the role of **RVJB59** in the stabilization of p53 might have a positive impact on the inhibition of angiogenesis through *IL8*.

**Figure 10:**
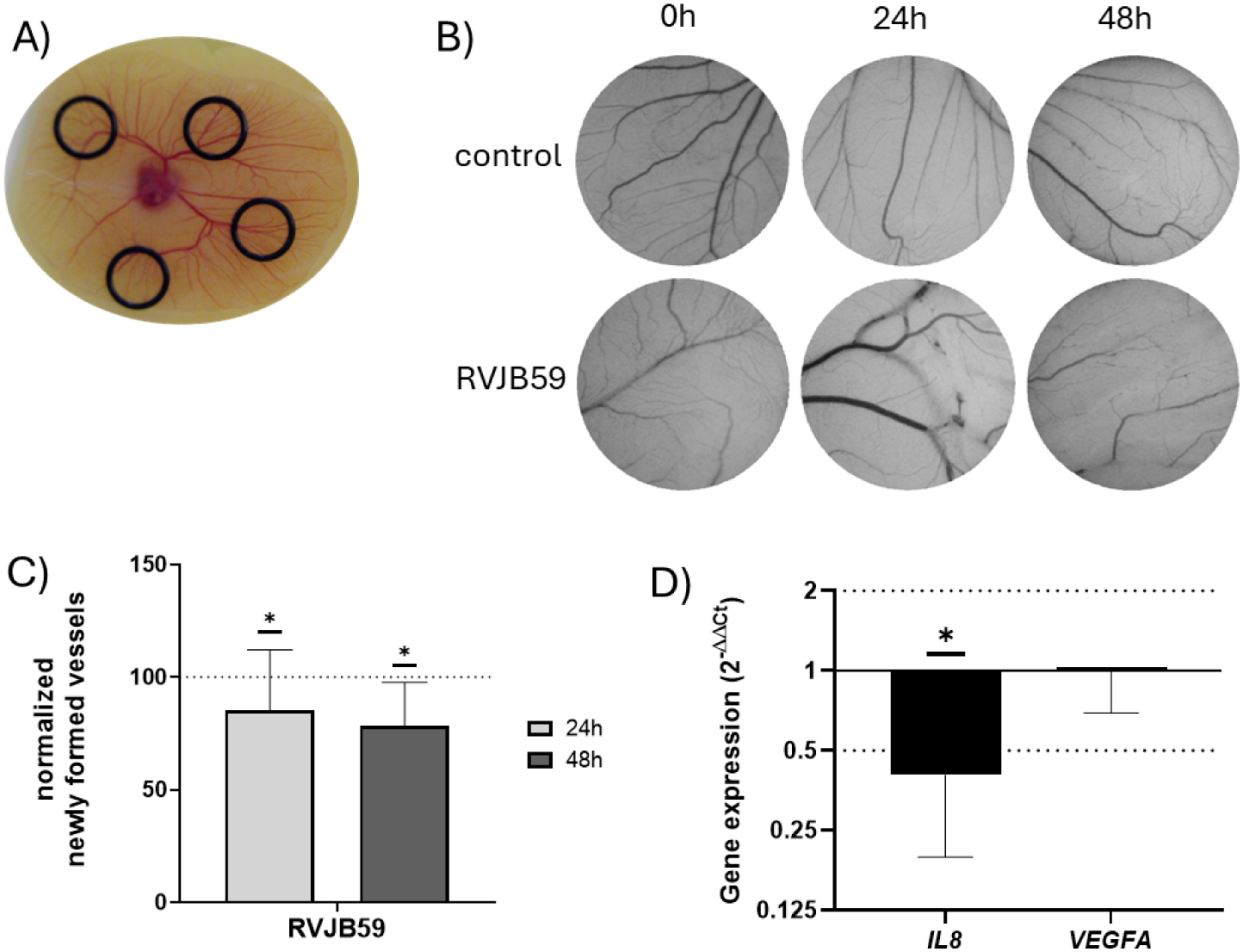
Ex-ovo chorioallantoic membrane (CAM) assay of a chicken embryo. (A) Aspect of the O-ring distribution in the embryo. (B) Aspect of the interior of the O-ring after addition of 21.1 μM of **RVJB59**, or 0.1% (v/v) DMSO (control). (C) Percentage of newly formed vessels after 24 or 48h exposure of the CAM to 21.1 μM of **RVJB59**. Bars represent the average ± standard deviation of at least 6 independent experiments, normalized to the number of tertiary veins after exposure to control and to the normalized number obtained in the corresponding CAM area at 0 h incubation. The dotted line at 100% represents the newly formed vessels of the control sample. (D) *IL8* and *VEGFA* gene expression after 18 h of incubation of the chicken embryo with 21.1 μM of **RVJB59**. Data were normalized to the GAPDH mRNA levels, followed by normalization to DMSO treated CAMs. Dotted lines at 2 and 0.5 represent the expression levels corresponding to overexpression and under-expression, respectively. *p-value <0.05.

## 3 Conclusions

In this work, we report on the study of the pharmacokinetic (PK) profile and the mechanism of action of tryptophanol-derived oxazoloisoindolinone **RVJB59**. The metabolic profile was assessed by LC-HRMS/MS in HLM and rat liver S9 fractions. Despite a slight decrease in metabolic stability compared to the p53 activator hits previously identified (**SLMP53–1** and **SLMP53–2**), **RVJB59** remains a low-clearance compound, presenting a half-life of 82 min (CLint 6.2 mL min^–1^ kg^–1^) in HLM and a half-life of 119 min (CLint 7.8 mL min^–1^ kg^–1^) in rat liver S9 fractions. Moreover, three main metabolites were identified, synthesized, and their antiproliferative activity tested in HCT116 colon cancer cells with and without p53. The three metabolites showed a decrease in activity compared with **RVJB59**, suggesting that this compound does not act as a pro-drug. Evaluation of the antiproliferative activity of **RVJB59** in a cancer cell line expressing R273H mutant p53 (HT29) and in fibroblasts indicated that this molecule is selective for cancer cells and may reactivate p53 in mutant p53 cells. To confirm if the mechanism of action involved the p53 target, the wt and R273H mut p53DBD thermostabilization by **RVJB59** was assessed by DSF. The compound was shown to stabilize the wt protein with an increment of the melting temperature of 1.43 °C, a value comparable to the one obtained with the positive control **MQ**. Furthermore, the DSF assay with R273H mut p53DBD showed melting curves with two melting transitions, different from the ones obtained for wt p53DBD. The susceptibility of the compound to undergo nucleophilic aromatic substitution by p53 protein was also assessed by HRMS/MS. The results showed that Cys141 of the wt p53DBD was the sole target for **RVJB59** covalent modification, thereby suggesting remarkable selectivity. Finally, the effect of **RVJB59** was also evaluated in 3D cells and in an *in vivo* assay with chicken embryos. **RVJB59** reduced 3D tumor spheroid growth and showed antiangiogenic potential, further highlighting the potential of **RVJB59** as a new anticancer drug through activation of p53. Future studies will focus on the detailed investigation of the interaction mechanism between **RVJB59** and the R273H mutant p53 protein, as well as evaluating the activity of **RVJB59** against other mutant p53 proteins.

## 4 Experimental Section

### 4.1 General Methods

All reagents were purchased from commercial suppliers and used without further purification unless otherwise indicated. The pET15b expression construct used to produce the human wt p53DBD was acquired from Addgene, and Sypro Orange was obtained from Invitrogen (5000× stock solution). Thrombin (Thrombin Restriction Grade 69671) was acquired from Merck Millipore. Analytical thin-layer chromatography was performed with silica gel plates (Merck, TLC Silica Gel 60 F254), and the spots were visualized using a UV lamp. Flash column chromatography was performed with Merck Silica Gel 60 (200–400 mesh). Melting points were obtained using a Kofler camera Bock monoscope M. The specific rotation values were determined with *p*-200 high-accuracy digital polarimeter (JASCO). ^1^H and ^13^C NMR spectra were recorded on a Bruker 400 MHz Avance II NMR spectrometer and a Bruker 500 MHz Avance II+ NMR spectrometer. ^1^H and ^13^C NMR chemical shifts are reported in parts per million (ppm, δ), referenced to the solvent used, and the proton coupling constants (*J*) are given in Hertz (Hz). Multiplicities are given as s (singlet), d (doublet), dd (double doublet), t (triplet), q (quartet), and m (multiplet). Spectra were assigned using appropriate COSY, APT, HMQC, and HMBC sequences. All compounds are >95% pure by HPLC analysis.

Plasma was obtained from healthy donors under a cooperative protocol established between the Instituto Português do Sangue e da Transplantação and Instituto Superior Técnico (IST), and received approval by the IST Ethic Committee (Reference number 26/2021).

### 4.2 Expression and Purification of Wild-Type p53 Protein

wt p53DBD (94–312) was cloned into the pET-15b expression vector (Novagen Inc.), which adds an N-terminal hexahistidine tag (wt 6xHis p53DBD), and was used to transform *E. coli* BL21­(DE3)­pLysS competent cells. Bacteria were grown in LB medium (500 mL) supplemented with zinc sulfate (final concentration: 100 μM) and ampicillin, at 37 °C with shaking. After reaching an OD of 0.6, protein expression was induced with IPTG (0.5 mM final concentration) at 27 °C overnight with shaking (140 U/min). Afterward, bacterial cells were pelleted by centrifugation (20 min at 4000 rpm, 4 °C) and lysed in cold lysis buffer (50 mM phosphate buffer, 300 mM NaCl, 10% glycerol , and pH 7.5) supplemented with lysozyme (1 mg/mL), PMSF (1 mM), DNase (5 μg), and TritonX-100 (0.8%). After incubation for 30 min on ice, the cells were lyzed by pulsed sonication (4 cycles of 60 s at 50% amplitude, with 30 s between cycle), then centrifuged (40 min at 10,000 rpm, 4 °C). The cellular lysate supernatant was collected and incubated on ice for 1 h with imidazole (10 mM), β-mercaptoethanol (10 μM), and Ni-NTA agarose. After incubation, samples were loaded into a 2 mL bed volume Poly-Prep chromatography column (Bio-Rad) and washed with increasing concentrations of imidazole (20 mM, 50 mM, and 75 mM). The wt 6xHis p53DBD was eluted using 250 mM imidazole, centrifuged (10 min at 10,000 rpm, 4 °C), and further purified by size exclusion chromatography (SEC). For this step, samples were loaded onto an SEC column (HiLoad 16/60 Superdex 200 prep grade) and purified over 3 h in SEC buffer (25 mM phosphate buffer, 150 mM NaCl, pH 7.4) using an ÄKTAprime plus FPLC system. The collected protein sample was quantified using the Bradford method, and the 6xHis tag was cleaved with thrombin at a 300 mU:30 μg (thrombin:p53DBD) ratio. Cleavage was performed in cleavage buffer (20 mM Tris-HCl, pH 8.4, 150 mM NaCl, and 2.5 mM CaCl_2_) supplemented with DTT (final concentration: 2 mM) and conducted overnight at 4 °C with stirring. Successful cleavage was confirmed by Western blot analysis using an anti-His antibody.

### 4.3 Mutagenesis for R273H Mutant p53DBD

cDNA necessary for mutagenesis with wt p53DBD was obtained from transformed competent cells following the ISOLATE II plasmid mini kit (Bioline) protocol. Primers necessary to produce mut R273H (forward: 5’-GCTTTGAGGTGCATGTTTGTG CC-3’; reverse: 5‘-GGCACAAACATGCACCTCAAAGC-3‘) were acquired from STAB VIDA, and mutagenesis was performed following the NZYmutagenesis kit protocol (from NZYtech), which involved PCR amplification followed by DpnI digestion (5 μL) for 1 h at 37 °C. Competent TOP10 *Escherichia coli* cells were transformed with 5 μL of the mutagenesis reaction. Five isolated colonies from each reaction were used to isolate the mutagenized DNA, which was sequenced. After confirmation of correct mutation introduction, the obtained DNA was used to further transform *Escherichia coli* BL21 (DE3) competent cells. Overnight preinoculum was then prepared for each mutant, and glycerol stocks were stored at −80 °C. The expression and IMAC purification protocol previously optimized for the wt p53DBD was used to obtain R273H p53DBD.

### 4.4 Cell Culture and Maintenance

All of the cell lines used in this study were acquired from ATCC (Manassas, Virginia, USA), except for the human colon adenocarcinoma p53-null isogenic derivative HCT116 p53^–/–^ cells, which were provided by B. Vogelstein (The Johns Hopkins Kimmel Cancer Center, Baltimore, MD, USA). HUVECs were maintained in F12-K medium (Thermo Fisher Scientific, Waltham, Massachusetts, USA) supplemented with 10% (v/v) fetal bovine serum (FBS, ATCC), 1 mg/mL heparin (Sigma-Aldrich, Merck, Darmstadt, Germany), 15 μg/mL endothelial cell growth factor (EGF, Sigma-Aldrich, Merck), and a mixture of 100 U/mL penicillin and 100 μg/mL streptomycin (Thermo Fisher Scientific). The other cell lines were maintained in Dulbecco’s modified eagle medium (DMEM, Thermo Fisher Scientific). Both media were supplemented with 10% (v/v) FBS (Thermo Fisher Scientific) and a mixture of 100 U/mL penicillin and 100 μg/mL streptomycin (Thermo Fisher Scientific). Cells were maintained, and all experiments were performed at 37 °C, 5% (v/v) CO2, and 99% (v/v) relative humidity.

### 4.5 Statistical Analysis

The data are presented as the mean and standard deviation from a minimum of three independent experiments. Statistical analysis was conducted using GraphPad Prism v8.2.1, and results were deemed statistically significant if the *p*-value was less than 0.05, as determined by the unpaired *t* test.

### 4.6 Synthesis

#### 4.6.1 Synthesis of (3*R*,9b*S*)-3-((1H-indol-3-yl)­methyl)-9b-(4-chloro-3-nitrophenyl)- 2,3-dihydrooxazolo­[2,3-*a*]­isoindol-5­(9bH)-one (**RVJB59**)

To a stirred solution of (*R*)-tryptophanol (1.02 g, 5.26 mmol) in toluene (50 mL) was added 2-(4-chloro-3-nitrophenyl)­benzoic acid (1.75 g, 5.78 mmol). The mixture was heated at reflux under a nitrogen inert atmosphere using Dean–Stark conditions for 16 h. The solvent was evaporated under reduced pressure, and the crude product was diluted in 15 mL of ethyl acetate and washed with a saturated aqueous solution of NaHCO_3_ (3 × 15 mL) and with brine (15 mL). The organic phase was dried in Na_2_SO_4_ and filtered. After evaporation of the solvent under reduced pressure, followed by purification using flash chromatography (eluent: ethyl acetate/*n*-hexane 4:6) and recrystallization in ethyl acetate/*n*-hexane, compound **RVJB59** was obtained as a yellow solid (1.85 g, 76%).^14^

#### 4.6.2 Synthesis of (3*R*,9b*S*)-9b-(4-chloro-3-nitrophenyl)-3-((2-oxoindolin-3-yl)­methyl)-2,3-dihydrooxazolo­[2,3-*a*]­isoindol-5­(9bH)-one (M1) and *N*-(2-(2-((3*R*,9b*S*)-9b-(4-chloro-3-nitrophenyl)-5-oxo-2,3,5,9*b*-tetrahydrooxazolo­[2,3-*a*]­isoindol-3-yl)­acetyl)­phenyl)­formamide (M2)

To a solution of **RVJB59** (101.5 mg, 219.7 μmol) and White–Chen catalyst (8) (10 mol%, 20.5 mg) in acetonitrile (5 mL), acetic acid (25.1 μL, 439.5 μmol) was added, followed by the addition of hydrogen peroxide (35 wt %) (8.5 μL, 98.8 μmol). The reaction mixture was stirred at 40 °C for 4 h. Then, a saturated aqueous solution of sodium bisulfite (5 mL) and ethyl acetate (2.5 mL) were added to the reaction mixture. The organic fraction was washed sequentially with an aqueous saturated solution of NaHCO_3_ (5 mL), water (5 mL), and brine (5 mL). The organic phase was dried over Na_2_SO_4_, filtered, and concentrated under reduced pressure. Compounds **M1** (9.8 mg, 23%), **M2** (14.3 mg, 33%), and unreacted **RVJB59** (61.3 mg) were isolated by preparative thin-layer chromatography (MeOH 2% in CH_2_Cl_2_).

#### 4.6.3 (3*R*,9b*S*)-9b-(4-chloro-3-nitrophenyl)-3-((2-oxoindolin-3-yl)­methyl)-2,3-dihydrooxazolo­[2,3-*a*]­isoindol-5­(9bH)-one (M1)

Duplication of NMR signals was observed due to the formation of two diastereomers (a and b are used to indicate their presence);^1^H NMR (500 MHz, Acetone-d_6_) δ*_H_* 9.44 (br s, 1H, NH), 8.39–8.38 (m, 2H, ArH a + b), 8.05 (dd, *J* = 8.3, 2.0 Hz, 1H, ArHa), 8.01 (dd, *J* = 8.4, 2.1 Hz, 1H, ArHb), 7.85–7.73 (m, 5H, ArH a + b), 7.68–7.61 (m, 4H, ArH a + b), 7.46 (d, *J* = 7.4 Hz, 1H, ArH a), 7.42 (d, *J* = 7.6 Hz, 1H, ArH b), 7.23–7.16 (m, 2H, ArH a + b), 7.13 (d, *J* = 7.1 Hz, 1H, ArH b), 7.02 (t, *J* = 7.3 Hz, 1H, ArH b), 6.96–6.89 (m, 3H, ArCH a + b), 4.81–4.66 (m, 4H, H3 a + b, OCH_2_ a/b), 4.01–3.93 (m, 2H, OCH_2_ a/b), 3.71–3.68 (m, 1H, CH a), 3.67–3.63 (m, 1H, CH b), 2.00–1.93 (overlapped with acetone-d_6_ signal, CH_2_ a), and 1.73–1.66 (m, 2H, CH_2_ b); ^13^C NMR (126 MHz, Acetone-d_6_) δ_C_ 179.5 (CH b), 179.0 (CH a), 175.1 (C=O), 149.4 (C–NO_2_ a + b), 147.2 (Cq b), 147.1 (Cq a), 143.6 (Cq b), 143.5 (Cq a), 141.9 (Cq b), 141.6 (Cq a), 134.8 (CH a), 134.7 (CH b), 133.4 (CH a), 133.2 (CH b), 132.10 (CH a), 132.05 (CH b), 131.74 (CH a), 131.69 (CH b), 131.64 (Cq b), 131.57 (Cq a), 130.9 (Cq a), 130.1 (Cq b), 128.73 (CH b), 128.68 (CH a), 127.1 (C–Cl a + b), 126.1 (CH a), 125.14 (CH a), 125.09 (CH b), 124.8 (CH b), 124.7 (CH a), 124.6 (CH b), 124.12 (CH a), 124.07 (CH b), 122.36 (CH a), 122.32 (CH b), 110.3 (CH. b), 110.2 (CH a), 100.6 (C-9b a + b), 76.9 (OCH_2_ a + b), 55.6 (C-3 b), 55.5 (C-3 a), 44.2 (CH a), 43.9 (CH b), 36.8 (CH_2_ a), and 36.1 (CH_2_ b). LC-ESI­(+)-MS/MS for C_25_H_18_ClN_3_O_5_ *m*/*z* 476.1019 [M + H]^+^. Found *m*/*z* 476.1007.

#### 4.6.4 *N*-(2-(2-((3*R*,9b*S*)-9b-(4-chloro-3-nitrophenyl)-5-oxo-2,3,5,9*b*-tetrahydrooxazolo­[2,3-*a*]­isoindol-3-yl)­acetyl)­phenyl)­formamide (M2)

^1^H NMR (500 MHz, Acetone-d_6_) δ_H_ 11.27 (br s, 1H, NH), 8.68 (d, *J* = 8.0 Hz, 1H, ArH), 8.57 (s, 1H, NCHO), 8.32 (d, *J* = 1.8 Hz, 1H, ArH), 7.95–7.92 (m, 2H, ArH), 7.77 (t, *J* = 8.0 Hz, 2H, ArH), 7.69–7.62 (m, 2H, ArH), 7.59 (t, *J* = 8.5 Hz, 1H, ArH), 7.45 (d, *J* = 6.4 Hz, 1H, ArH), 7.17–7.14 (m, 1H, ArH), 4.89 (m, 1H, OCH_2_), 4.76–4.68 (m, 1H, H-3), 4.09 (m, 1H, OCH_2_), 3.74 (dd, *J* = 17.4, 4.5 Hz, 1H, CH_2_), and 3.27 (dd, *J* = 17.4, 9.4 Hz, 1H, CH_2_); ^13^C NMR (126 MHz, Acetone-d_6_) δ_C_ 197.1 (C=O), 174.3 (C=O), 161.4 (C=O), 149.4 (C-NO_2_), 147.0 (Cq), 141.5 (Cq), 140.5 (Cq), 135.6 (CH), 134.8 (CH), 133.3 (CH), 132.2 (CH), 132.1 (CH), 131.8 (CH), 131.6 (Cq), 128.3 (Cq), 127.0 (Cq), 125.2 (CH), 124.7 (CH), 124.1 (CH), 123.7 (CH), 121.8 (CH), 100.3 (C-9b), 77.3 (OCH_2_), 53.7 (C-3), and 45.5 (CH_2_). LC-ESI­(+)-MS/MS for C_25_H_18_ClN_3_O_6_ *m*/*z* 492.0959 [M + H]^+^. Found *m*/*z* 492.0944.

#### 4.6.5 Synthesis of (3*R*,9b*S*)-3-((1*H*-indol-3-yl)­methyl)-9b-(3-amino-4-chlorophenyl)-2,3-dihydrooxazolo­[2,3-*a*]­isoindol-5­(9b*H*)-one (M3)

To a solution of *(R)*-tryptophanol (225.3 mg, 1.18 mmol) in toluene (10 mL) was added 2-(3-amino-4-chlorobenzoyl)­benzoic acid (361.2 mg, 1.31 mmol). The mixture was heated at reflux under a nitrogen inert atmosphere using Dean–Stark conditions for 16 h. The solvent was evaporated under reduced pressure. The crude product was dissolved in ethyl acetate (15 mL) and washed with a saturated aqueous solution of NaHCO_3_ (3 × 15 mL) and with brine (15 mL). The organic phase was dried over Na_2_SO_4_, filtered and concentrated under reduced pressure. Compound **M3** was obtained as a white solid (0.387 g, 76%) after flash chromatography (eluent ethyl acetate/*n*-hexane 1:1), followed by recrystallization in CH_2_Cl_2_/*n*-hexane. Mp: 84–86 °C; ${[\alpha ]}_{D}^{20}$ = −67.9° (*c* = 0.40, CH_2_Cl_2_).^1^H NMR (400 MHz, Acetone-d_6_) δ_H_ 10.06 (br s, 1H, NH), 7.74 (d, *J* = 6.2 Hz, 1H), 7.55 (m, 3H), 7.39 (d, *J* = 8.0 Hz, 1H), 7.35–7.25 (m, 3H), 7.20 (s, 1H), 7.11 (t, *J* = 7.4 Hz, 1H), 7.03 (t, *J* = 7.3 Hz, 1H), 6.92 (d, *J* = 8.2 Hz, 1H), 5.10 (br s, 2H, NH_2_), 4.67–4.60 (m, 1H, H-3), 4.49–4.45 (m, 1H, OCH_2_), 4.02–3.98 (m, 1H, OCH_2_), 3.19 (dd, *J* = 13.5, 5.7 Hz, 1H, CH_2_), and 2.78 (dd, *J* = 13.5, 9.7 Hz, 1H, CH_2_); ^13^C NMR (101 MHz, acetone-d_6_) δ_C_ 174.6 (C=O), 148.2 (Cq), 145.6 (Cq), 140.1 (Cq), 137.6 (Cq), 134.1 (CH), 131.8 (Cq), 131.0 (CH), 130.3 (CH), 128.4 (Cq), 124.7 (CH), 124.3 (CH), 123.7 (CH), 122.2 (CH), 119.6 (CH), 119.1 (CH), 118.9 (Cq), 115.7 (CH), 113.6 (CH), 112.2 (CH), 111.7 (Cq), 101.3 (C-9b), 76.8 (OCH_2_), 56.8 (C-3), and 31.1 (CH_2_). LC-ESI­(+)-MS/MS for C_25_H_20_ClN_3_O_2_ *m*/*z* 430.1317 [M + H]^+^. Found *m*/*z* 430.1328.

#### 4.6.6 Reaction of RVJB59 with Glutathione (GSH)

To a solution of compound **RVJB59** in acetonitrile (10.65 μmol, 4.9 mg in 200 μL, 5.33 × 10^–^ ^2^ M) reduced l-glutathione (13.37 mg, 42.62 μmol) dissolved in 200 μL of ABIC buffer (50 mM, pH 7.4) was added. The reaction mixture was incubated for 24 h at 37 °C and monitored by LC-ESI-HRMS/MS. A kinetic assay was conducted in similar conditions, using **MQ** and **acrylamide** as positive controls, and a glutathione excess of 1000 eq. Aliquots were collected at distinct time points, quenched upon iodoacetamide addition, and monitored by HRMS/MS.

### 4.7 Differential Scanning Fluorimetry Assay

5 μg of wt p53DBD protein or R273H mut p53DBD protein were incubated with 1, 2, and 2.5 mM of the compound in SEC buffer (25 mM phosphate buffer, 150 mM NaCl, pH 7.4) with 1 mM TCEP. 5x Sypro Orange was added to each mixture (0.08 μL). Samples were placed in a 96-well plate (50 μL per well), and the plates were centrifuged (5 min at 300 rpm) before being analyzed. Fluorescence was assessed by Bio-Rad CFX96 Touch Real-Time PCR Detection System (Bio-Rad Laboratories, CA) at increasing temperatures from 20 to 90 °C, with a rate of 0.2 °C per minute, following 20 min of incubation at 20 °C. Tm values were calculated by boltzmann sigmoidal regression using GraphPad Prism 8 version 8.4.2 (GraphPad Software Inc., La Jolla, CA).

### 4.8 HRMS Protein Modification Assay

wt p53DBD was desalted against 20 mM ammonium bicarbonate buffer (pH 8.0) using a 10 kDa protein concentrator. 60 μg of wt p53DBD was treated with 50 μM MQ or **RVJB59** in 20 mM ABIC buffer for 2 h at room temperature (100 μL incubation volume). Samples were then precipitated with cold acetone (4 times the reaction volume) at 0 °C overnight. The acetone was removed, and the resulting pellets were resuspended in 50 mM ABIC buffer at pH 7.4, enriched with 7 M urea. TCEP was added to achieve a final concentration of 1 mM, and samples were incubated for 45 min at 37 °C with stirring. After this incubation , iodoacetamide (20 mM) was added, and samples were incubated again for 30 min in the dark with stirring. Then, samples were diluted with 50 mM ABIC buffer to get a final urea concentration of 1 M . The samples were then digested with trypsin (1:20 w/w) for 3 h. After this period, the reaction was stopped by the addition of formic acid (10% v/v) and stored in dry ice. Finally, samples were evaporated in a SpeedVac concentrator, and the resulting pellets were resuspended in 40 μL of acetonitrile/water (95:5, supplemented with 0.1% formic acid), centrifuged (5 min at 13000 rpm). The peptides were analyzed by liquid chromatography (Ultra High Performance Liquid Chromatography system, Bruker Elute, Mannheim, Germany) interfaced with a Bruker Impact II quadrupole time-of-flight mass spectrometer equipped with an electrospray source (Bruker Daltoniks, Mannheim, Germany). Chromatographic separation was performed on an Acclaim PepMap C18 column (1.0 mm × 150 mm, 3 μm particle size; Thermo Scientific). The mobile phase consisted of water containing 0.1% formic acid (A) and acetonitrile containing 0.1% formic acid (B). The elution conditions were as follows: 0.2% B for 1 min, 0.2–46.2% B over 59 min, 46.2–90% B over 1 min, 90% B for 4 min, 90–0.2% B over 1 min, and 0.2% B for 14 min. The injection volume was 10 μL, the flow rate was 100 μL·min^–^,^1^ and the column was maintained at 40 °C. Sample analysis was performed by data-dependent acquisition (auto MSMS mode) in the 300–2200 *m*/*z* range with a 2 Hz rate and by a dynamic method with a fixed cycle time of 3 s. The MS source parameters were set as follows: dry gas heater temperature, 200 °C; dry gas flow, 8 L·min^–^;^1^ and capillary voltage, 4500 V.

#### 4.8.1 HRMS Data Processing

Data were analyzed using MaxQuant version 2.0.1.0 (Copyright Max-Planck-Institute of Biochemistry) and Bruker DataAnalysis version 4.1 (Copyright 1993–2012 Bruker Daltonik GmbH). Search parameters included precursor ion mass tolerance of 10 ppm, fragment ion mass tolerance of 20 ppm, number of missed cleavages, ≤2, and variable amino acid modifications: oxidation of methionine, carbamidomethylation of cysteines, and **RVJB59** and **MQ** incorporation (mass increments of 424.13 and 138.09 Da, respectively) into cysteines. Additional research was carried out to investigate covalent modifications in other nucleophilic residues (e.g., Hist and Lys). The acquired MS/MS spectra were searched against a database containing only the human p53 protein sequence, obtained from UniProt. All spectra corresponding to **RVJB59-** and **MQ**-modified peptides were manually checked.

### 4.9 SRB Assay

Human colon adenocarcinoma (HCT116) cell lines were seeded in 96-well plates at a density of 5.0 × 10^3^ cells per well for 24 h. Cells were treated with serial dilutions of compounds (ranging from 3 to 150 μM) for an additional 48 h. Effects on cell proliferation were measured by sulforhodamine B (SRB) assay as previously described,^41^ and IC_50_ values were determined for each cell line using the GraphPad Prism software version 7.0 (La Jolla, CA, USA).

### 4.10 MTS Assays

HT29 and fibroblast cell lines were seeded in 96-well plates at an initial cell density of 7500 cells/well and allowed to adhere for 24 h. The medium was then replaced by crescent concentrations of **RVJB59** or DMSO, and the viability was evaluated after 48 h using the CellTiter 96 Aqueous One Solution Cell Proliferation Assay (Promega, Madison, Wisconsin, USA) according to manufacturer’s instructions. The percentage of cell viability was calculated after normalization to the corresponding viability of cells exposed to the same percentage DMSO.

### 4.11 Invasion Assay

For the invasion assay, HCT116 spheroids were prepared as previously described^34,42^ and after 4 days, 50 μL of medium was replaced by 50 μL of high-concentration Matrigel basement membrane (Corning, Merck) mixed 1:1 with 21.1 μM of **RVJB59**, 20 ng/mL EGF (positive control), or 0.4% (v/v) DMSO (negative control). After 1 h incubation at 37 °C, 21.1 μM of **RVJB59**, 20 ng/mL EGF, or 0.1% (v/v) DMSO was added on top of the respective spheroid in Matrigel. Images were acquired with a Ti–U Eclipse inverted microscope (Nikon Instruments, Japan) every 24 h for 7 days. The diameter of the spheroid was measured using ImageJ software.

#### 4.11.1 Determination of Spheroid Growth Rate

HCT116 and HCT116 – Fibroblasts were prepared as previously described^34^ and after 4 days, 50 μL of medium was replaced by 50 μL of medium supplemented with 21.1 μM of **RVJB59**, 20 ng/mL EGF (positive control), or 0.1% (v/v) DMSO (negative control). The size of the spheroid was measured every 24 h by acquiring images with a Ti–U Eclipse inverted microscope (Nikon) and ImageJ software, and the growth rate was measured by considering the size of the spheroids during the following 4 days after adding the compound.

### 4.12 Wound Healing Assay

For the wound healing assay, cells were seeded in 24-well plates containing DMEM supplemented with 10% FBS and an antibiotic mixture. After 24 h, a pipette tip was used to scratch the cell monolayer, and the medium was replaced with fresh medium containing either 21.1 μM of **RVJB59** or 0.1% (v/v) ethanol (vector control). The scratch was monitored for 24 h using a Cytosmart Lux2 (Axion Biosystems, Atlanta, GA, USA), and the percentage of wound closure was calculated by measuring the scratch size at 0 h and after 24 h using ImageJ software.

### 4.13 Angiogenic Tube Formation

The *in vitro* angiogenesis evaluation followed a previously described protocol.^36^ Initially, wells of a 96-well culture plate were coated with 50 μL of high-concentration Matrigel basement membrane (Corning, Merck) and incubated for 30 min at 37 °C. HUVECs at 80% confluence in a T25 flask were then detached using TrypLE Express (ThermoFisher Scientific), and a mixture was prepared with 1.5 × 10^5^ cells/mL and 21.1 μM of **RVJB59**, or 0.1% (v/v) DMSO, all in F12–K medium supplemented with 5% (v/v) FBS, 0.5 mg/mL heparin, 15 μg/mL ECGF, and 0.5% (v/v) antibiotic/antimycotic mixture. This mixture was gently applied over the matrix, and images were captured every 15 min for 24 h using a Cytosmart Lux2 microscope and associated software (Axion Biosystems, Atlanta, GA, USA).

### 4.14 Ex-Ovo Chorioallantoic Membrane (CAM) Assay of the Chicken Embryo

The experiments were conducted using previously described procedures.^38,43^ In brief, after 72 h of incubation at 38 °C and 90% relative humidity, fertilized eggs (Pinto Valouro, Bombarral, Portugal) were opened in a weighing boat, ensuring the embryo faced upward. Black silicone O-rings (inside diameter: 8 mm) were placed equidistantly above the blood vessels of the embryo, and 40 μL of samples were added to the O-rings, ensuring that each embryo was exposed to 0.1% DMSO (control) or 21.1 μM of **RVJB59**. The percentage of newly formed vessels was calculated as described before.^37^

Chicken embryo mRNA analysis was performed according to procedures previously described by our group.^38,44^ Each embryo was exposed to three O-rings containing the same stimulus, 0.1% (v/v) DMSO or 21.1 μM of **RVJB59**. The expression of *IL8* and *VEGFA* genes was analyzed using the 2^–ΔΔCt^ method, with *GAPDH* as the internal control and *VEGFA* as the control.

## Supplementary Material



## Notes

Mass spectra of **RVJB59**, **M1**, **M2**, and **M3** with the respective fragmentation patterns, depletion plot obtained in HLM and rat S9 liver homogenate with **RVJB59**, thermal denaturation curve obtained with **MQ**, proton NMR and carbon NMR of **M3**, and full scan and tandem HRMS/MS spectra of identified modified peptides (PDF)

## Abbreviation

ABIC: ammonium bicarbonate
CAM: chorioallantoic membrane
DMEM: Dulbecco’s Modified Eagle medium
DMSO: dimethyl sulfoxide
DSF: differential scanning fluorimetry
DTT: dithiothreitol
FPLC: fast protein liquid chromatography
GSH: glutathione
HLM: human liver microsomes
HRMS: high-resolution mass spectrometry
HUVEC: human umbilical vein endothelial cells
IL8: interleukin 8
IPTG: isopropyl β-d-1-thiogalactopyranoside
LB: lysogeny broth
MDM2: mouse double minute 2
MDM4: mouse double minute 4
PBS: phosphate-buffered saline
PMSF: phenylmethylsulfonyl fluoride
p53 DBD: p53 DNA-binding domain
SEC: size exclusion chromatography
TCEP: tris­(2-carboxyethyl)­phosphine
VEGFA: vascular endothelial growth factor A
wt: wild-type
