# Supplementary material for: Mechanistic Insights of a p53-Targeting Small Molecule
Source: ACS Pharmacol Transl Sci. 2025 May 7;8(6):1726–40. doi: 10.1021/acsptsci.5c00110 (PMC12171893; doi:10.1021/acsptsci.5c00110)
Supplement: Supplementary file 1 [file pt5c00110_si_001.pdf]

Supporting information

Mechanistic insights of a p53 targeting small molecule

Ricardo J. F. Ferreira,[a,1] Valentina Barcherini,[a,1] Catarina Roma-Rodrigues,[b,c,d] Miriama Sikorová,[b,c] Lucília Saraiva,[e] Ana P. Leandro,[a] Pedro V. Baptista, [b,c] Alexandra R. Fernandes, [b,c] Alexandra M. M. Antunes[f,] Maria M. M. Santos[a,*]*

a) Research Institute for Medicines (iMed.ULisboa), Faculty of Pharmacy, Universidade de Lisboa, Av. Prof. Gama Pinto, 1649-003 Lisboa, Portugal

b) Associate Laboratory i4HB—Institute for Health and Bioeconomy, NOVA School of Science and Technology, NOVA University Lisbon, 2819-516 Caparica, Portugal

c) UCIBIO—Applied Molecular Biosciences Unit, Department of Life Sciences, NOVA School of Science and Technology, NOVA University Lisbon, 2819-516 Caparica, Portugal

d) BIORG—Bioengineering and Sustainability Research Group, Faculdade de Engenharia, Universidade Lusófona, 1749-024 Lisboa, Portugal

e) LAQV/REQUIMTE, Laboratory of Microbiology, Department of Biological Sciences, Faculty of Pharmacy, University of Porto, Rua de Jorge Viterbo Ferreira 228, Porto, 4050-313, Portugal

f) Centro de Química Estrutural (CQE), Institute of Molecular Sciences, Departamento de Engenharia Química, Instituto Superior Técnico (IST), Universidade de Lisboa, 1049-001 Lisboa, Portugal

1 – These authors contributed equally to this work.

*Corresponding authors: mariasantos@ff.ulisboa.pt for M. M. M. Santos; alexandra.antunes@tecnico.ulisboa.pt for A. M. M. Antunes

Contents

Figure S1. Depletion plot of compound **RVJB59** in human liver microsomes. 3
Figure S2. Depletion plot of compound **RJVB59** in rat S9 liver homogenate. 3
Figure S3. Tandem high resolution mass spectrum obtained through ESI-(+) of protonated molecule observed at m/z 460.1077, which corresponds to compound **RJVB59**, and proposed fragmentation pattern. 4
Figure S4. Tandem high resolution mass spectrum of protonated molecule observed at m/z 476.1019, which corresponds to a 16u-mass increment when compared to protonate molecule of parent compound **RJVB59**. Proposed fragmentation pattern. 4
Figure S5. Tanden high resolution mass spectrum and proposed fragmentation pattern obtained for metabolite **M2**. 5
Figure S6. Tandem high resolution mass spectrum obtained by ESI-(+) for the protonated molecule observed at m/z 430.1328. Proposed structures for the fragment ions observed in the tandem mass spectrum. 5
Figure S7. Thermal denaturation curves obtained by DSF assay of the wt p53DBD in the absence and presence of **MQ** (2.0 mM). The DSF assays were performed with the fluorophore SYPRO Orange. 6
Figure S8. Tandem high resolution mass spectra obtained by ESI-(+) for the protonated molecule observed at m/z 731.2179 ppm and proposed fragmentation mechanism. 6
Figure S9. Proton NMR of **M3**. 7
Figure S10. Carbon APT NMR of **M3**. 7
Figure S11. **A**. Full scan and **B**. Tandem HRMS/MS spectrum of the tetra charged ion corresponding to ^{111}LGFLHSGTAKSVTCTYSPALNK^{132} peptide, bearing **MQ** modification at Cys124. 8
Figure S12. **A**. Full scan and **B**. Tandem HRMS/MS spectrum of the tri charged ion corresponding to ^{140}TCPVQLWVDSTPPPGTR^{156} peptide, bearing **MQ** modification at Cys141. 8
Figure S13. **A**. Full scan and **B**. Tandem HRMS/MS spectrum of the tricharged ion corresponding to ^{140}TCPVQLWVDSTPPPGTR^{156} peptide, bearing **RVJB59** modification at Cys141. 9
Figure S14. **A**. Full scan and **B**. Tandem HRMS/MS spectrum of the dicharged ion corresponding to ^{140}TCPVQLWVDSTPPPGTR^{156} peptide, bearing **RVJB59** modification at Cys141. 9

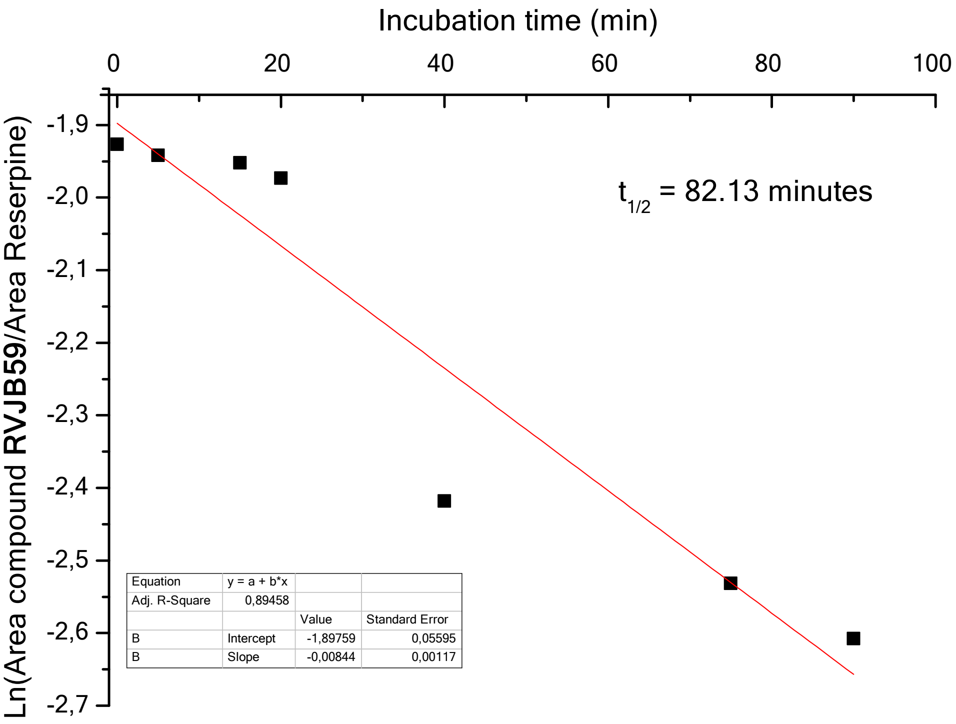

Figure S1. Depletion plot of compound **RVJB59** in human liver microsomes.

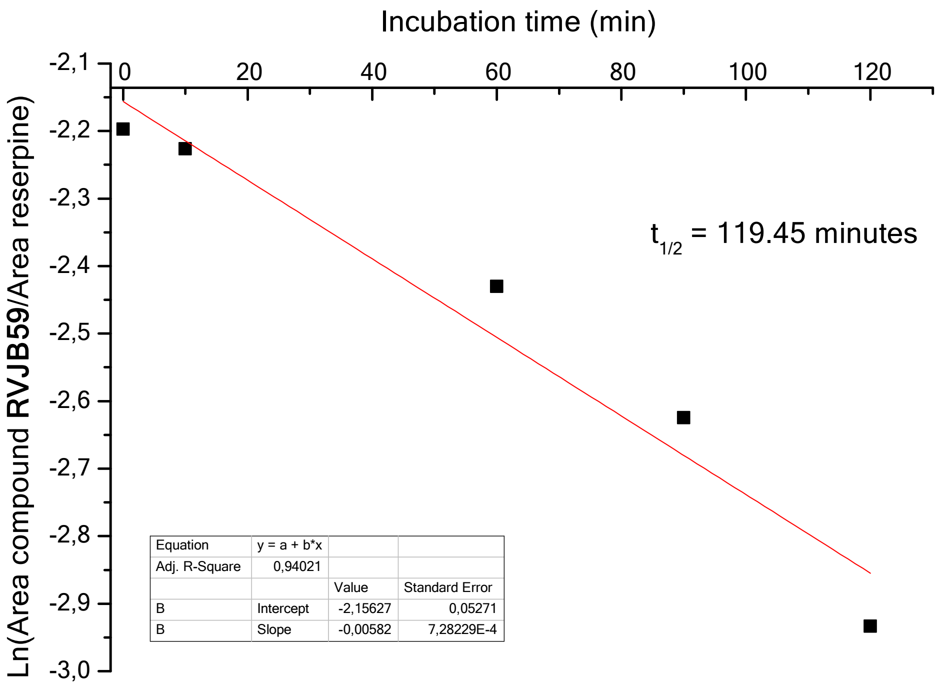

Figure S2. Depletion plot of compound **RJVB59** in rat S9 liver homogenate.

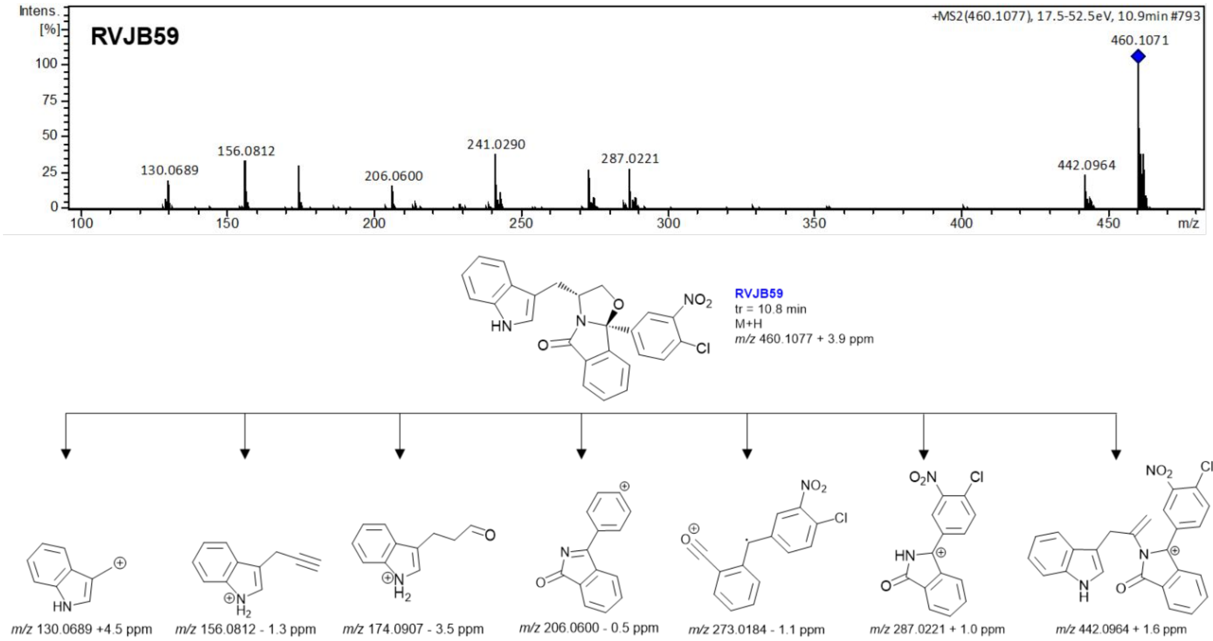

Figure S3. Tandem high resolution mass spectrum obtained through ESI-(+) of protonated molecule observed at m/z 460.1077, which corresponds to compound **RJVB59**, and proposed fragmentation pattern.

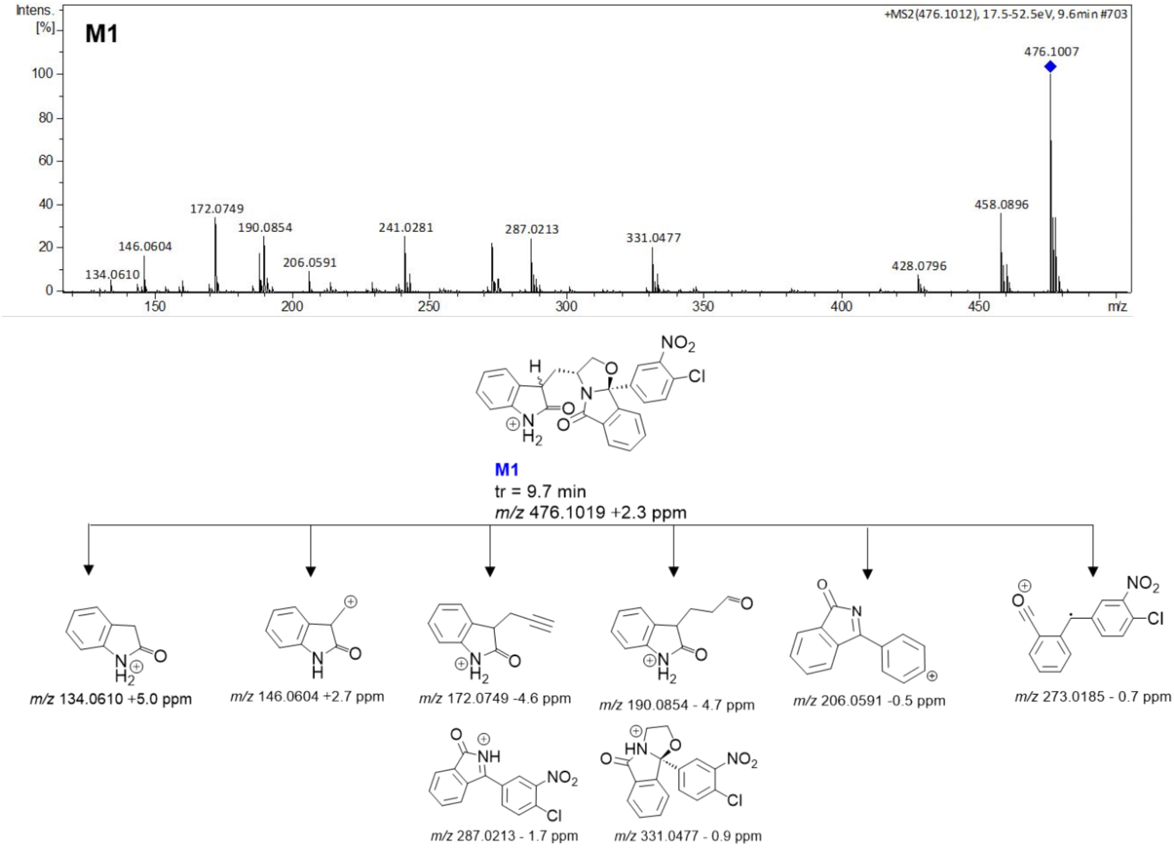

Figure S4. Tandem high resolution mass spectrum of protonated molecule observed at m/z 476.1019, which corresponds to a 16u-mass increment when compared to protonate molecule of parent compound **RJVB59**. Proposed fragmentation pattern.

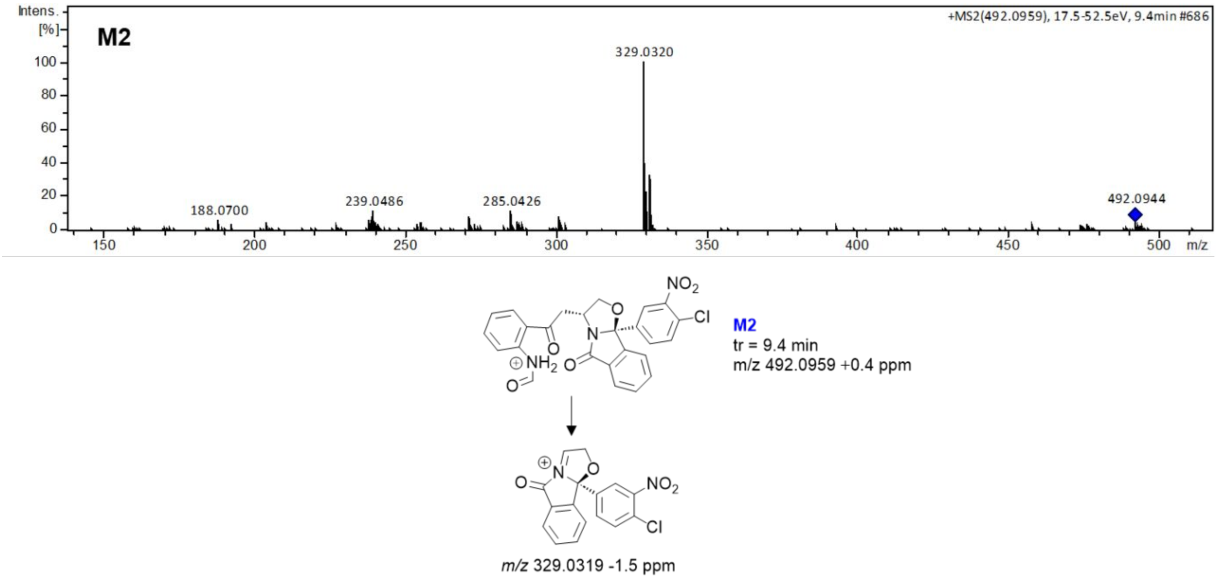

Figure S5. Tanden high resolution mass spectrum and proposed fragmentation pattern obtained for metabolite **M2**.

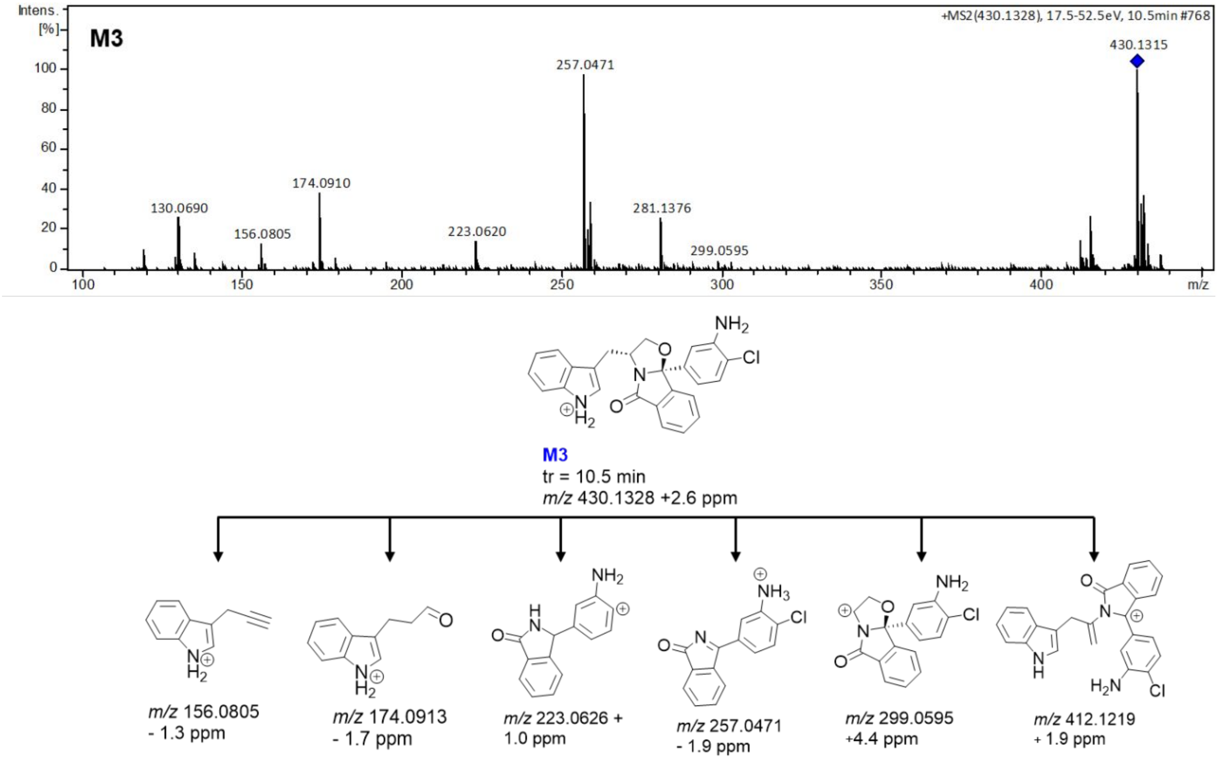

Figure S6. Tandem high resolution mass spectrum obtained by ESI-(+) for the protonated molecule observed at m/z 430.1328. Proposed structures for the fragment ions observed in the tandem mass spectrum.

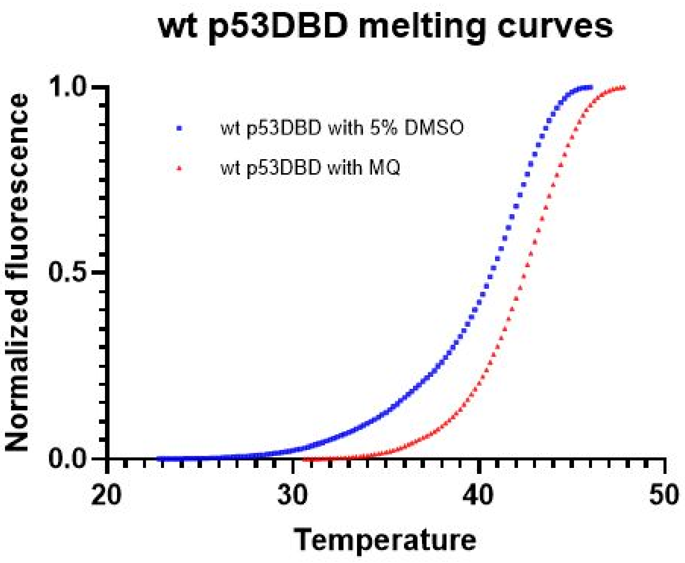

Figure S7. Thermal denaturation curves obtained by DSF assay of the wt p53DBD in the absence and presence of **MQ** (2.0 mM). The DSF assays were performed with the fluorophore SYPRO Orange.

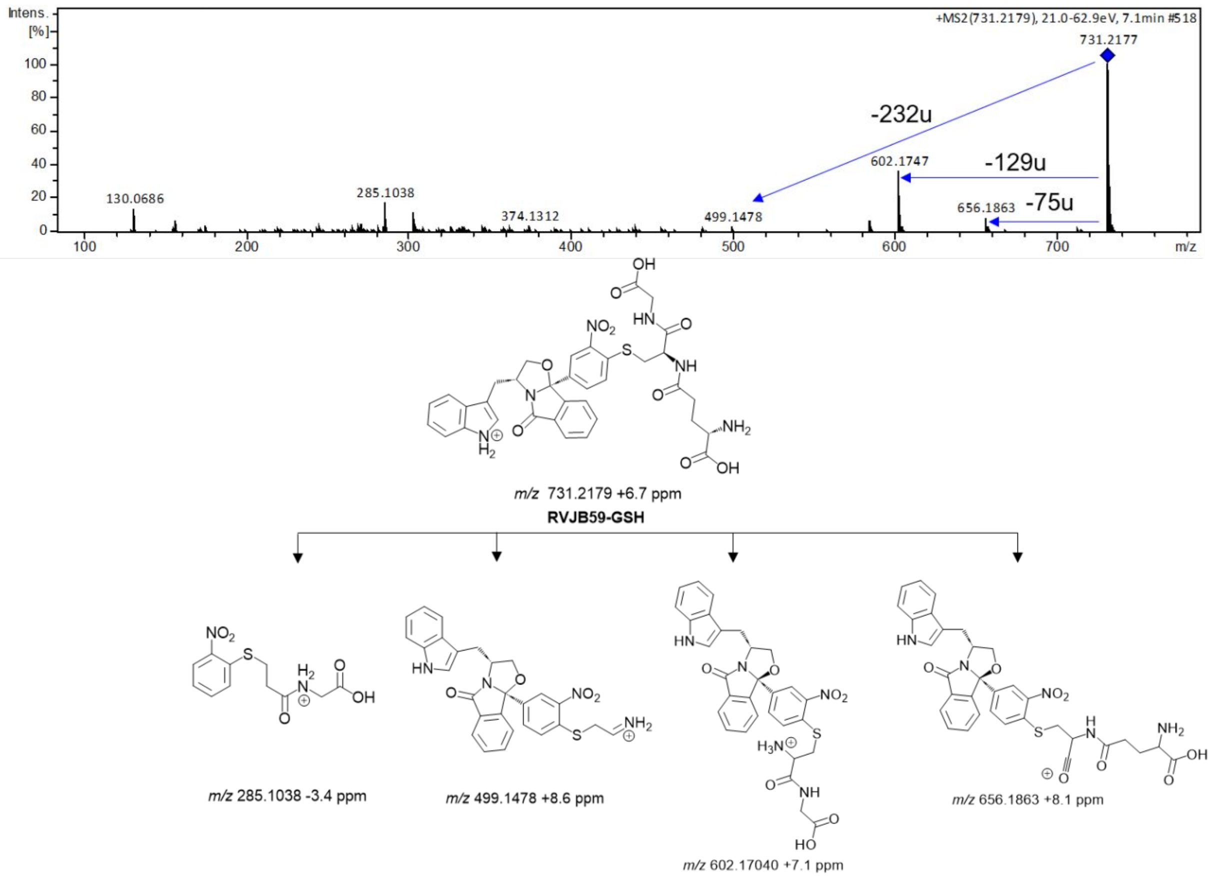

Figure S8. Tandem high resolution mass spectra obtained by ESI-(+) for the protonated molecule observed at m/z 731.2179 ppm and proposed fragmentation mechanism.

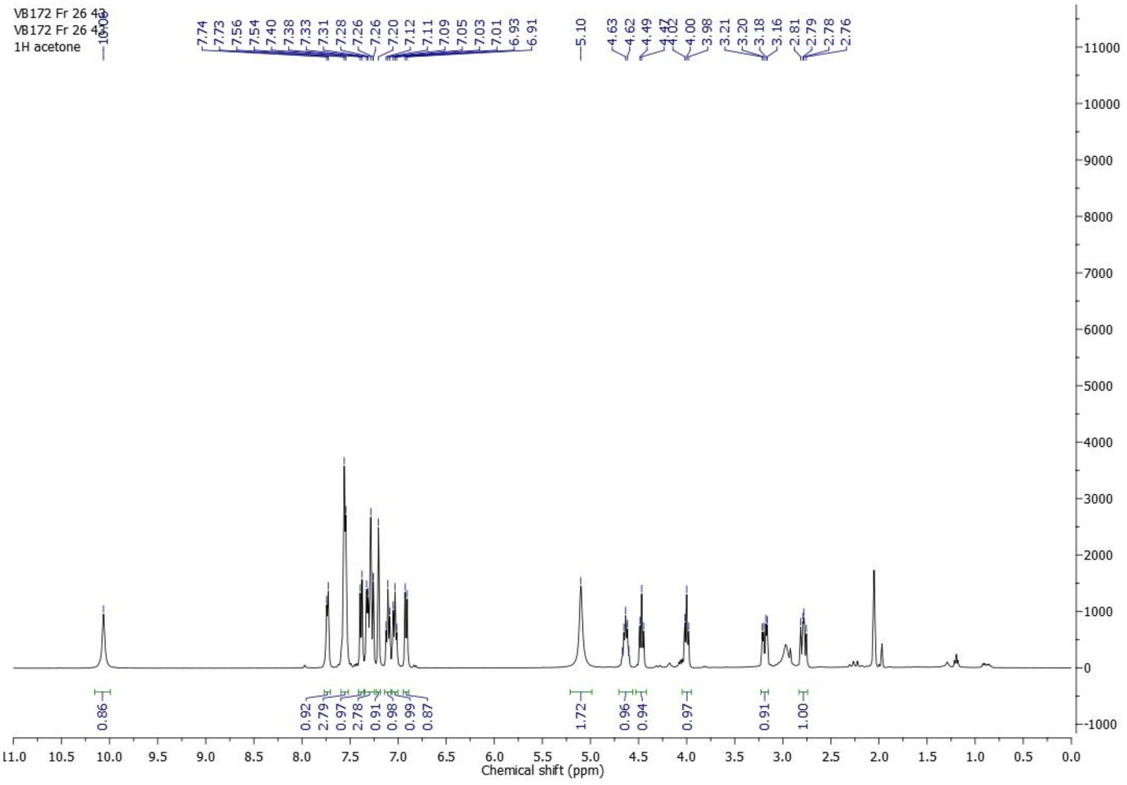

Figure S9. Proton NMR of **M3**.

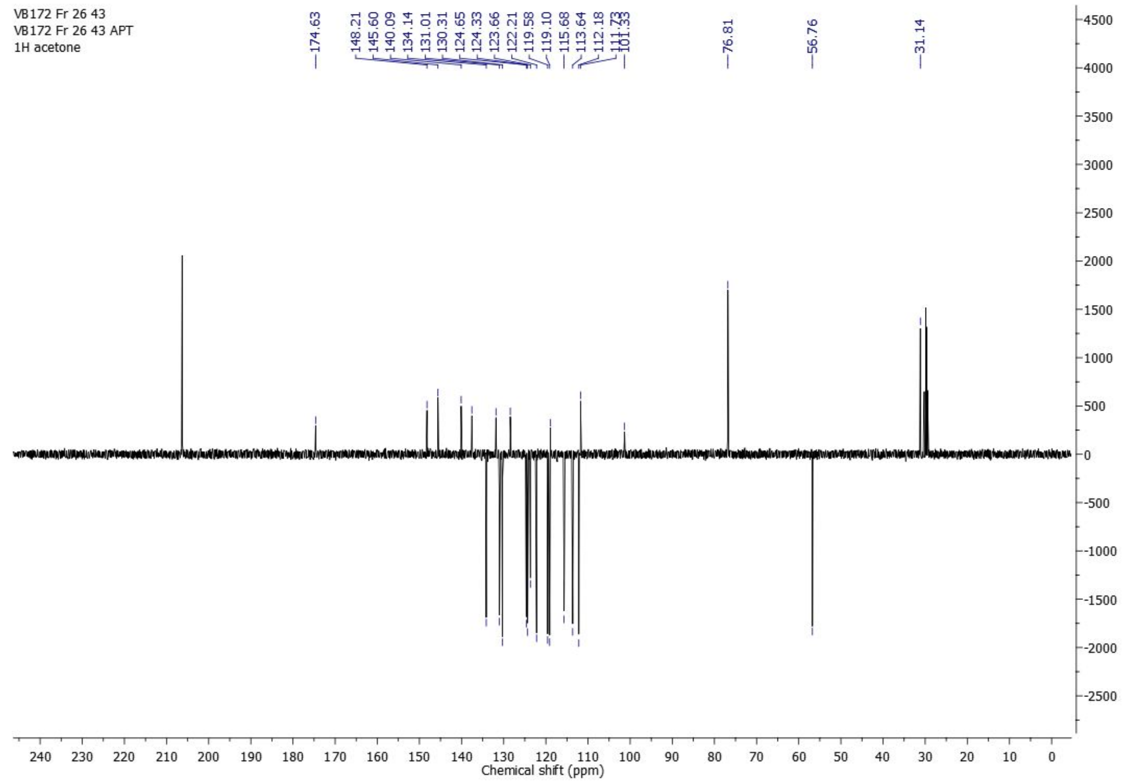

Figure S10. Carbon APT NMR of **M3**.

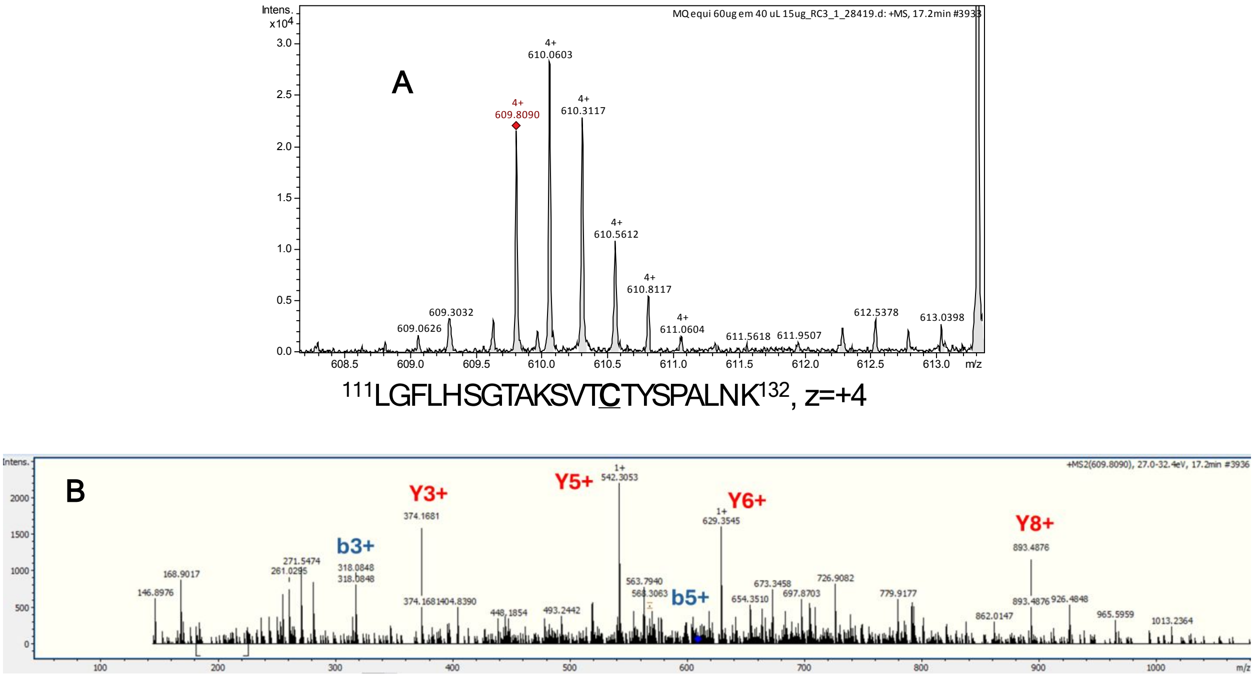

Figure S11. **A**. Full scan and **B**. Tandem HRMS/MS spectrum of the tetra charged ion corresponding to [111]LGFLHSGTAKSVTCTYSPALNK[132] peptide, bearing **MQ** modification at Cys124.

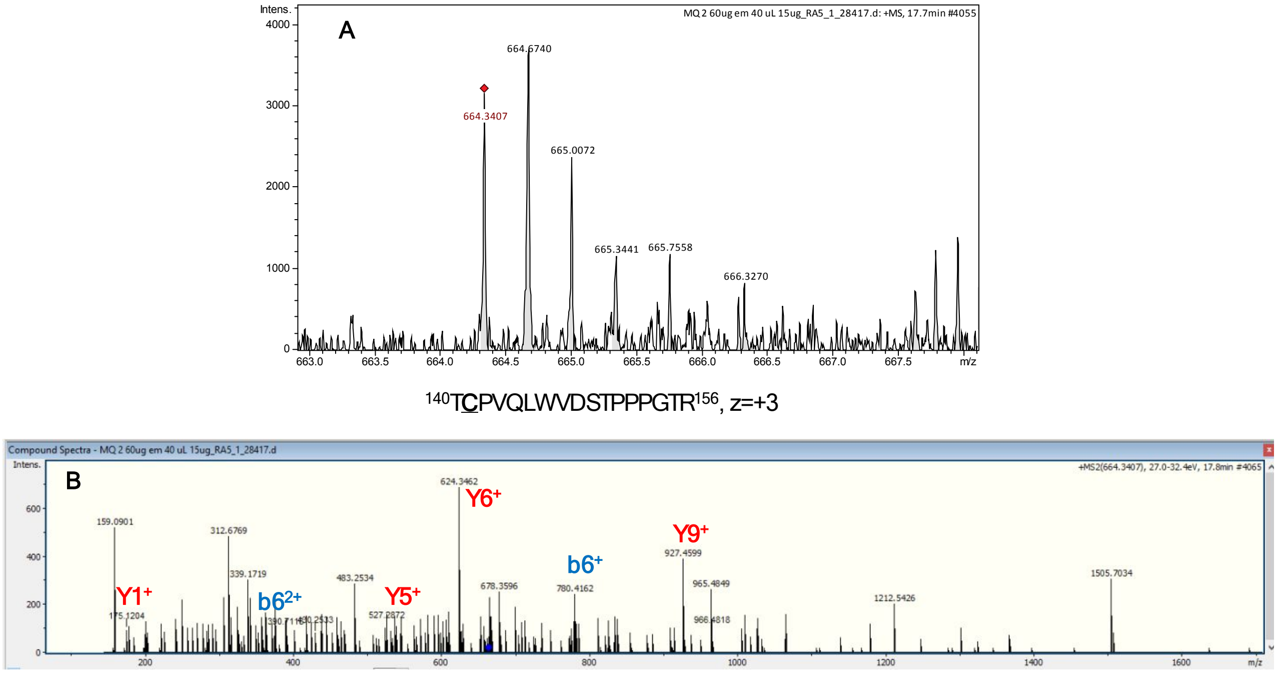

Figure S12. **A**. Full scan and **B**. Tandem HRMS/MS spectrum of the tri charged ion corresponding to [140]TCPVQLWVDSTPPPGTR[156] peptide, bearing **MQ** modification at Cys141.

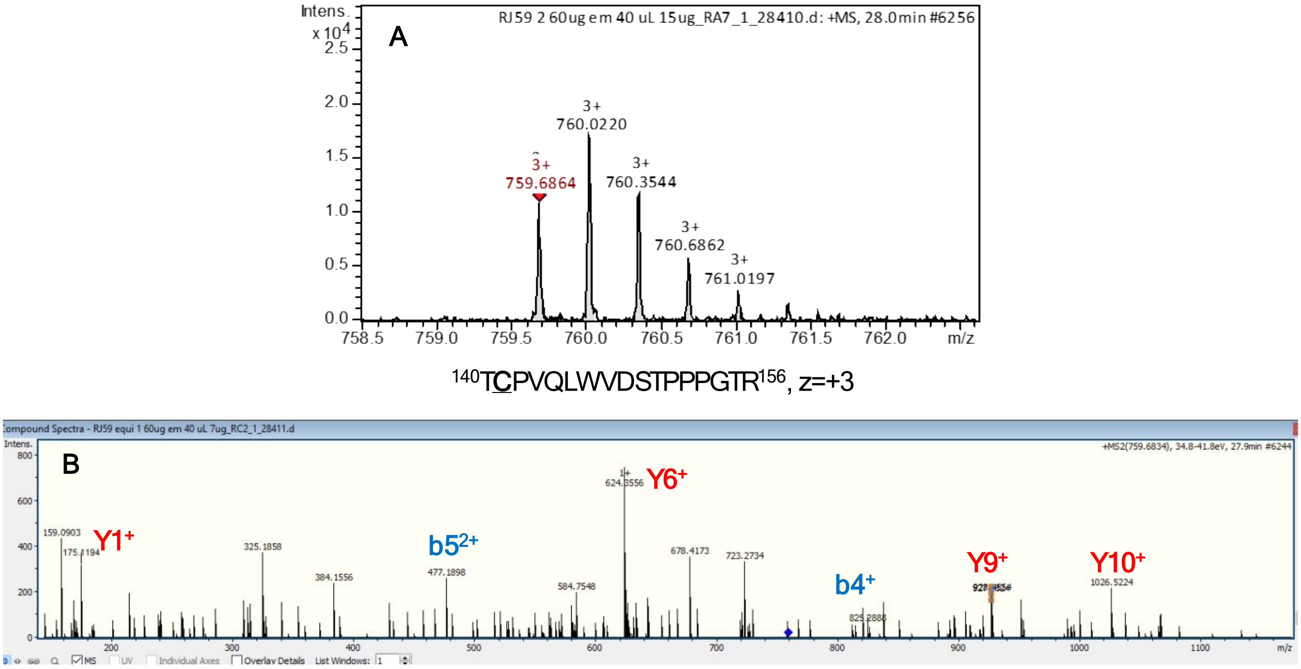

Figure S13. **A**. Full scan and **B**. Tandem HRMS/MS spectrum of the tricharged ion corresponding to [140]TCPVQLWVDSTPPPGTR[156] peptide, bearing **RVJB59** modification at Cys141.

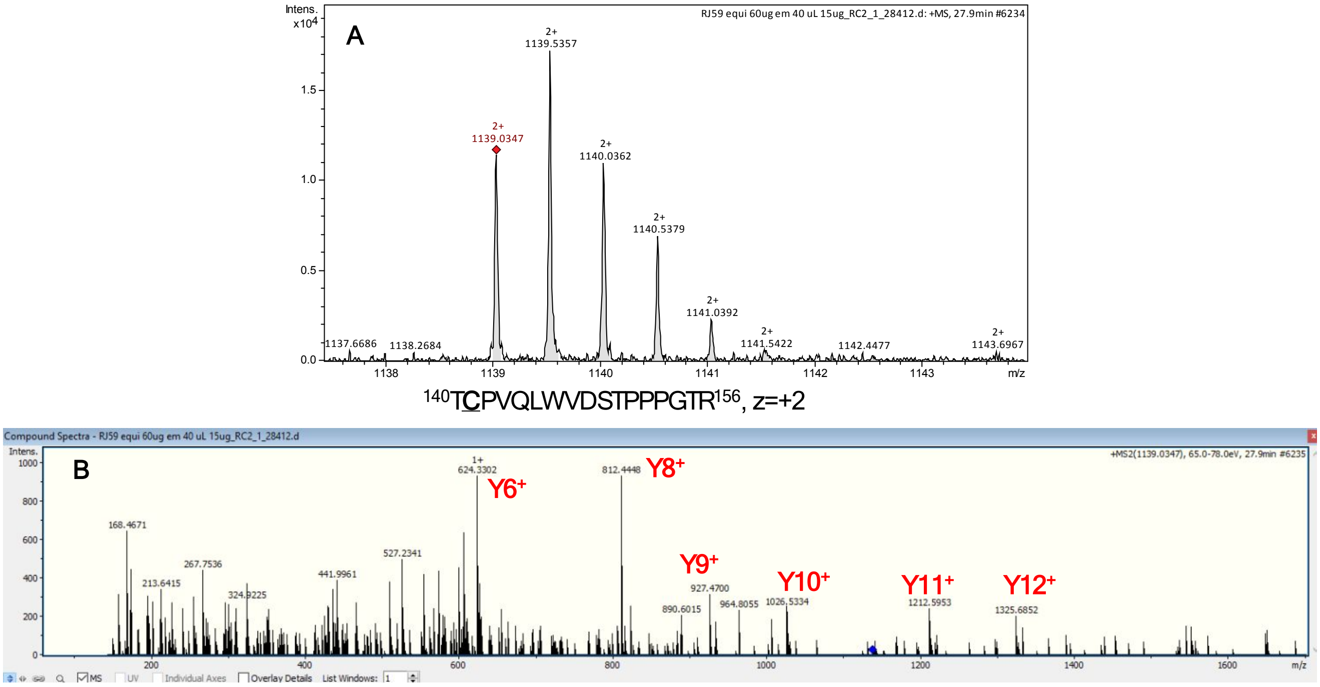

Figure S14. **A**. Full scan and **B**. Tandem HRMS/MS spectrum of the dicharged ion corresponding to [140]TCPVQLWVDSTPPPGTR[156] peptide, bearing **RVJB59** modification at Cys141.
